# Regulation of the DEAH/RHA helicase Prp43 by the G-patch factor Pfa1

**DOI:** 10.1073/pnas.2203567119

**Published:** 2022-11-21

**Authors:** Marieke Enders, Ralf Ficner, Sarah Adio

**Affiliations:** ^a^Department of Molecular Structural Biology, Institute of Microbiology and Genetics, Georg-August-University Göttingen, D-37077 Göttingen, Germany

**Keywords:** DEAH/RHA helicases, G-patch proteins, ribosome biogenesis, splicing, molecular machines

## Abstract

The DEAH/RHA helicase Prp43 remodels protein–RNA complexes during pre-messenger RNA (mRNA) splicing and ribosome biogenesis. The helicase activity and ATP turnover are intrinsically low and become activated by G-patch (gp) factors in the specific cellular context. The gp motif connects the helicase core to the flexible C-terminal domains, but it is unclear how this affects RecA domain movement during catalysis and the unwinding of RNA substrates. We developed single-molecule Förster Resonance Energy Transfer (smFRET) reporters to study RecA domain movements within Prp43 in real time. Without Pfa1(gp), the domains approach each other adopting predominantly a closed conformation. The addition of Pfa1(gp) induces an open state, which becomes even more prevalent during interaction with RNA. In the open state, Prp43 has reduced contacts with bound nucleotide and shows rapid adenosine diphosphate (ADP) release accelerating the transition from the weak (ADP) to the strong (apo) RNA binding state. Using smFRET labels on the RNA to probe substrate binding and unwinding, we demonstrate that Pfa1(gp) enables Prp43(ADP) to switch between RNA-bound and RNA-unbound states instead of dissociating from the RNA. ATP binding to the apo-enzyme induces the translocation along the RNA, generating the unwinding force required to melt proximal RNA structures. During ATP turnover, Pfa1(gp) stimulates alternating of the RecA domains between open and closed states. Consequently, the translocation becomes faster than dissociation from the substrate in the ADP state, allowing processive movement along the RNA. We provide a mechanistic model of DEAH/RHA helicase motility and reveal the principles of Prp43 regulation by G-patch proteins.

## Significance

DEAH/RHA helicases remodel RNP complexes in central processes of RNA metabolism such as transcription, splicing, or translation at the cost of adenosine triphosphate (ATP) hydrolysis. These enzymes require activation to function efficiently in their specific cellular context. We show that G-patch factors do not only recruit the DEAH/RHA helicase Prp43 to its cellular localization, but also interfere with its mechanism of motion along RNA substrates. Instead of dissociating from the substrate after a single round of catalysis, Prp43 bound to G-patch translocates continuously along the RNA, resolving stable RNA structures. Our research reveals the mechanism of RNA duplex unwinding by Prp43 and demonstrates how this process is regulated by its cellular cofactors.

RNA helicases of the DEAH/RHA family are involved in key steps of RNA metabolism and reorganize structured RNAs and RNA–protein complexes (RNPs) through ATP-dependent translocation in 3′ to 5′ direction along single strands (1). They share conserved sequence motifs (I, Ia, Ib, II, III, IV, V, and VI) with the helicase superfamily 2, which spread over two RecA domains that together form the helicase core (2). These essential motifs are responsible for ATP and RNA binding, ATP hydrolysis, and the coupling between hydrolysis and unwinding (2–4). Further domains include a common set of C-terminal domains composed of a winged helix (WH), a helix bundle (HB, previously called ratchet-like domain), and an oligosaccharide binding (OB) -fold domain as well as varying N-terminal extensions (5, 6). RNA is bound in a sequence-independent manner in a channel between the RecA and the C-terminal domains (7–12). A mechanistic model for single strand RNA (ssRNA) translocation has been developed from crystal structures of DEAH/RHA helicases in different conformational states (7–10, 13, 14): Transitions between open, nucleotide-free and closed, nucleotide-bound conformations of the helicase core lead to alternate accommodation of either four or five RNA nucleotides stacked in the binding channel with a conserved loop in RecA1 and a β-hairpin in RecA2 serving as “bookends” preventing slippage. Thereby, translocation in 3′ to 5′ direction with a step size of 1 nt per hydrolyzed ATP is achieved. In vivo targets of DEAH/RHA helicases often lie buried within large RNPs and are not accessible by direct translocation (15–18). In a mechanism described as winching, it was proposed that the force generated by translocation is transmitted to the interior of the RNP disrupting RNA structures distant from the helicase binding site (19). Nevertheless, the precise mechanism linking translocation and the unwinding of duplex RNA structures is still unknown.

Some specialized DEAH-box helicases target their substrates with high sequence specificity, such as DHX36, which binds RNA- and DNA- quadruplex structures with high affinity (13, 20). However, the vast majority of DEAH-box helicases bind RNA with low intrinsic specificity and employ auxiliary protein factors for the recruitment to their cellular place of action (21–23). Prp43 and its human homolog DHX15 are such ubiquitous DEAH-box helicases which are activated in different functional contexts by so-called G-patch (gp) factors, named after a 40–50 amino-acid-long glycine-rich consensus motif (24, 25). In yeast, Prp43 interacts with four different G-patch partners. Ntr1 recruits Prp43 to the spliceosome where it mediates the disassembly of the intron lariat complex in late states of splicing (16, 26, 27) and where it disrupts stalled spliceosome complexes associated with suboptimal or mutated pre-mRNA substrates (28, 29). Together with Pfa1, Prp43 promotes 20S to 18S ribosomal RNA (rRNA) processing during the maturation of 40S ribosome subunits (30, 31), while the G-patch protein Gno1 links Prp43 to precursors of the 60S ribosome promoting the release of small nucleolar RNAs from their pre-mRNA binding sites (14, 32, 33). In the cytoplasm, Prp43 interacts with the G-patch protein Cmg1, but the function and target of this complex remain to be identified (34). Functions of the human DHX15 protein additionally include RNA unwinding during mRNA capping and transcription regulation (35, 36).

So far, eight of the more than 20 human G-patch factors are known to regulate DHX15 activity; however, the interaction partners of most remaining G-patch factors are still undiscovered (25). Besides the G-patch motif, these factors share little similarity regarding size, structure, and domain organization except for the high prevalence of RNA binding domains linking them to the various pathways of RNA metabolism (25). However, there is mounting evidence that they employ a common mechanism regulating the activity of Prp43/DHX15.

G-patch factors enhance the ATPase and helicase activities of Prp43/DHX15; interestingly, the G-patch motif alone is sufficient for efficient stimulation. (7, 14, 31, 32, 36–39). In the spliceosome, the isolated G-patch motifs Ntr1(gp) and even Pfa1(gp) can replace full-length Ntr1 during Intron Lariat Spliceosome (ILS) disassembly (27, 29). These findings demonstrate that the G-patch motif primarily stimulates Prp43 activity in a specific cellular context rather than guiding Prp43 to its binding site. The G-patch motif is intrinsically disordered and only forms secondary structure elements upon contact with a helicase (37, 40). In the crystal structure of the DHX15-NKRF(gp) complex, the G-patch gp-motif forms a flexible brace between the RecA2 and WH domain in DHX15 reaching across the back side of the RNA binding channel (41). The overall conformation strongly resembles the RNA-bound structure of Prp43 in complex with ADP-BeF_3_^2−^ with closed RecA domains (7). Based on these findings, the authors proposed that G-patch binding stabilizes a compact state of the helicase competent of ATP turnover and with a tight grip on the RNA substrate (41). Comparison of the DHX15–NKRF(gp) complex with the scPrp43–Ntr1 complex in the ILS (16) shows a similar binding mode of the gp motif to the helicase suggesting that activation occurs by comparable mechanisms. It is important to notice that the scPrp43–Ntr1 complex in the ILS shows open RecA domains, whereas in the DHX15–NKRF(gp) complex, the RecA domains are in a closed state (16, 41).

Overall, the molecular mechanism of helicase stimulation by G-patch factors remains unclear. Open questions concern how G-patch factors alter structure and function of Prp43 during ATP turnover and how G-patch-dependent changes affect Prp43 motility on the RNA. We use single molecule Förster Resonance Energy Transfer (smFRET) to monitor in real-time how the G-patch motif affects the conformational cycling of Prp43 RecA domains. Using smFRET labels on the RNA substrate, we follow RNA binding and unwinding by the Prp43-G-patch complex. We show that the G-patch motif induces the open state of RecA domains which is incompatible with nucleotide binding and facilitates nucleotide release. We confirm that the G-patch motif enhances RNA binding and demonstrate that ATP binding generates sufficient force to melt short RNA duplexes. During turnover, the G-patch accelerates transitions of the RecA domains between the open and closed state which couples ATP hydrolysis to processive motion along the RNA. Our results reveal the principal regulation mechanism of Prp43 by G-patch partners and lead to a mechanistic model of its motility cycle.

## Results

### Pfa1(gp) Stabilizes the Open Conformation of Prp43 RecA Domains.

**Fig. 1.**
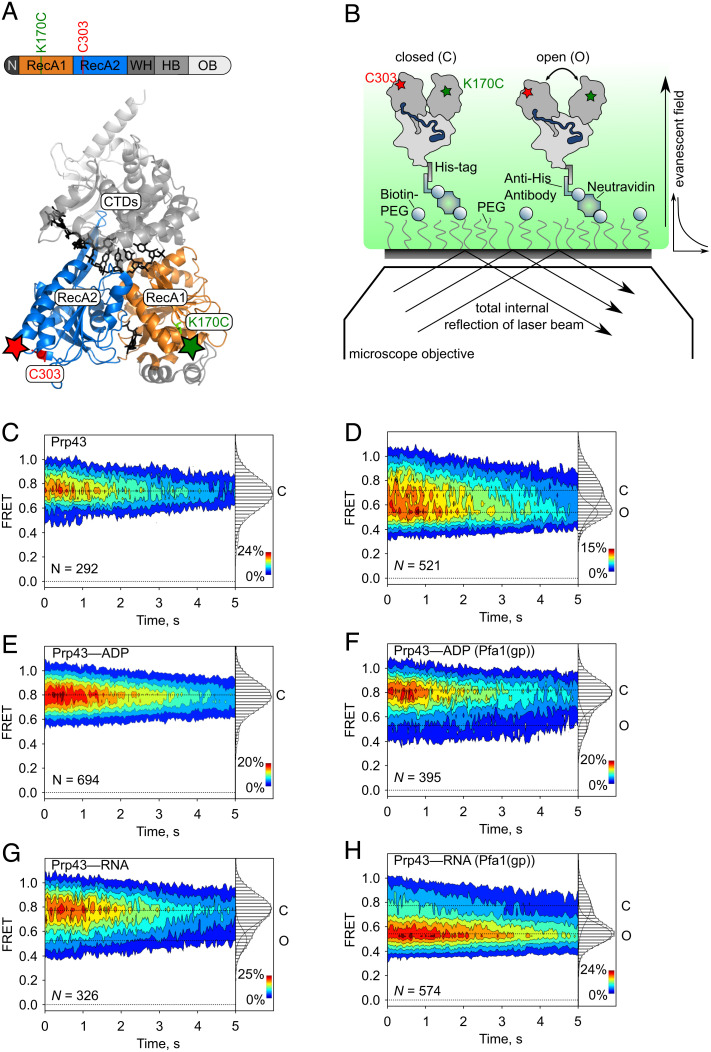
Pfa1(gp) promotes opening of RecA domains in Prp43. (*A*) Domain organization and structure of ctPrp43 (residues 61–764) with U_7_-RNA and ADP-BeF_3_^−^ (Protein Data Bank (PDB) ID code 5lta). Remainders of the N-terminal domain (residues 61–96) and the C-terminal domains (winged-helix: residues 459–526, helix-bundle: residues 527–640, and OB-fold: residues 641–764) are shown in grey shades; RecA1 (residues 97–273) and RecA2 (residues 274–458) domains are shown in orange and blue, respectively. RNA and ADP-BeF_3_^–^ are shown in black. Cy3- and Cy5-label positions at K170C and C303 are indicated as red and green stars. (*B*) TIRF microscopy experiment scheme monitoring RecA domain movement in Prp43. The C-terminal His-tag of Prp43 interacts with a biotinylated antibody for attachment on neutravidin-functionalized coated coverslips. Distance changes between Cy3 and Cy5 report on the conformation (closed or open) of the helicase core. (*C*–*H*) Contour plots and 2D histograms showing the distribution of FRET values (mean ± SD) of (*C*) Prp43 in the apo state (0.77 ± 0.03), (*D*) Prp43–Pfa1(gp) complex (0.75 ± 0.05 and 0.56 ± 0.04), (*E*) Prp43–ADP (0.81 ± 0.02), (*F*) Prp43–ADP–Pfa1(gp) complex (0.82 ± 0.03 and 0.54 ± 0.03), (*G*) Prp43–RNA (0.79 ± 0.02 and 0.53 ± 0.04), and (*H*) Prp43–RNA–Pfa1(gp) complex (0.77 ± 0.02 and 0.54 ± 0.01) corresponding to closed (C) or open (O) conformations. Contour plots show that traces are stable over time and last for several seconds. Normalization was performed here and in all further FRET distributions by the number of FRET counts. *N* is here and all further plots the number of individual traces. Data are from N = 3 independent experiments.

To analyze domain movements in Prp43 by smFRET, we generated fluorescence labeling sites at position C170 in RecA1 and C303 in RecA2 of *Chaetomium thermophilum* Prp43 (ctPrp43_Cys_) and a control mutant with labels at C187 in RecA1 and C303 in RecA2 (ctPrp43_Cys_2) (Fig. 1*A* and *SI Appendix*, Fig. S1A and *Methods*).

We validated that rates of ATP turnover by labeled Prp43 variants and functional Prp43 mutants in the absence and presence of saturating concentrations of ctPfa1(gp) and ssRNA are similar to wild-type ctPrp43(wt) and to scPrp43 indicating that the catalytic activity is not impaired by the fluorescence tags (*SI Appendix*, Fig. S1 *B* and *C*). We immobilized Prp43 on streptavidin-functionalized glass cover slips (42) using a biotin-tagged antibody against the C-terminal His-tag and followed FRET on individual Prp43 molecules at steady state conditions by total internal reflection of fluorescence (TIRF) microscopy (Fig. 1*B*). In the absence of ligands, Prp43 shows a single population of signals with E_FRET_ = 0.77 (Fig. 1*C* and *SI Appendix*, Fig. S2*A* and Table S1), corresponding to an estimated donor–acceptor distance of 4.3 nm. This value is comparable to the distance between the C_α_ atoms of the labeled residues in the crystal structure of ctPrp43–ADP where the RecA domains are close together (4.2 nm, PDB: 5d0u (43), *SI Appendix*, Table S2). We denominate this conformation the closed (C) state. Next, we followed smFRET of the Prp43–Pfa1(gp) complex in order to test whether the G-patch alters the conformation of RecA domains in Prp43 (Fig. 1*D*). In addition to the C state, we observe a second population of signals with lower FRET efficiency (E_FRET _= 0.56, *SI Appendix*, Fig. S2*B* and Table S1) indicating the opening of the RecA domains in Prp43 in response to Pfa1(gp) binding. We denominate this conformation the open (O) state. O and C states are nearly equally populated (*SI Appendix*, Table S1), and a small fraction of smFRET traces (7%) shows transitions between the states suggesting slow exchange between the populations (*SI Appendix*, Fig. S2 *C* and *D*). In the presence of ADP, Prp43 and the Prp43–Pfa1(gp) complex show similar FRET states compared to the apo-enzyme (Fig. 1 *E* and *F* and *SI Appendix*, Table S1). However, for the G-patch complex, the C state is more prevalent in the presence of ADP, which agrees with the notion that contacts between RecA domains and the nucleotide stabilize the C conformation (41). Interestingly, when bound to ssRNA, Prp43 can access the O state in the absence of Pfa1(gp), while the conformation of the RecA domains in the Prp43–Pfa1(gp)–RNA complex is almost entirely shifted toward the O state (Fig. 1 *G* and *H*). Similar results were obtained with the control Prp43_Cys_2. Prp43–ADP shows a single population of signals with E_FRET _= 0.53 corresponding to the C state (*SI Appendix*, Fig. S3 *A* and *B* and Tables S1 and S2). The Prp43–Pfa1(gp)–RNA complex shows a second population with E_FRET _= 0.40 corresponding to the O state and the distribution of populations is comparable to Prp43_Cys_ (*SI Appendix*, Fig. S3 *C* and *D* and Tables S1 and S2). Hence, the opening of the Prp43 RecA domains is principally possible through binding of the RNA substrate, similar as for other DEAH-box helicases such as DHX36 or Prp22 (10, 13). The binding of Pfa1(gp) alters the conformational distribution of Prp43 such that the C state becomes less prominent (Fig. 1*H*).

### Pfa1(gp) Promotes ADP Release by Prp43.

To explore the implications of the open RecA domains on Prp43 function, we compared the position of the C_α_ atoms of the labeled residues with their equivalents in DEAH/RHA helicase crystal structures with open and closed RecA domains (*SI Appendix*, Table S2). We used the structure of ctPrp43–ADP (PDB: 5d0u (43)) as example for closed RecA domains and the structures of the scPrp43–Ntr1 complex in the spliceosomal context (PDB: 5y88 (16)) and of the highly homologous DEAH-box helicase ctPrp22 in complex with a ssRNA (PDB: 6i3p (10)) as examples for open RecA domains.

**Fig. 2.**
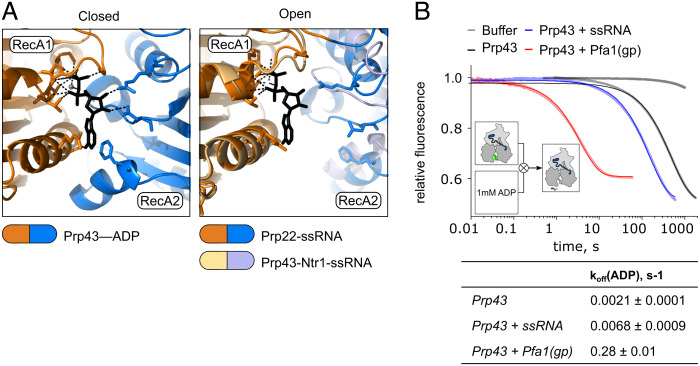
The opening of RecA domains facilitates ADP release. (*A*) Modeling of ADP binding by Prp43 with closed and open RecA domains. *Left* panel: Nucleotide binding site of ctPrp43–ADP in the closed state ((43); PDB 5d0u), where ADP is sandwiched between RecA1 (orange) and RecA2 (blue). The adenine moiety (black) is bound by π–π electron stacking with F360 in the RecA2 and R162 in the RecA1 domain. The ribose is bound by hydrogen bonding with D391 and R435 in Rec2 and phosphates hydrogen bond with G122, G124, K125, T126, and T127 in RecA1. *Right* panel: Overlay of the nucleotide binding site of scPrp43 as part of the intron-lariat spliceosome ((16); PDB: 5y88, pale orange and light blue) and the closely related ctPrp22 bound to U_12_-RNA ((10); PDB: 6i3p, orange and blue) with open RecA domains. The position of ADP was derived by alignment of RecA1 domains in open and closed state structures, assuming that contacts with RecA1 are maintained during domain opening. RecA2 is too far distant to maintain the interactions with ADP. Due to limited resolution, the scPrp43 structure does not contain side chains. Therefore, the residues that interact with ADP in the closed state structure are only shown for ctPrp22. (*B*) Release of mant-ADP from Prp43 monitored by stopped-flow apparatus. Experiment scheme: Syringe 1 contains Prp43 preincubated with mant-ADP, where mant fluorescence (bright green) is induced by tryptophane residues acting as FRET donor. Syringe 2 contains 1 mM unlabeled ADP. Upon rapid mixing the release of mant-ADP becomes apparent by loss of mant fluorescence. Averages of fluorescence time traces, normalized by fluorescence intensity, are shown for Prp43, Prp43–ssRNA, and Prp43-Pfa1(gp). Table indicates mant-ADP dissociation rates (k_off_(ADP) ± SD) derived from at least N = 3 independent measurements.

In the open state structures, the RecA domains were about 0.8 nm further apart from each other than in the closed state structures, which agrees with the distance change of approximately 1 nm derived from the smFRET data (*SI Appendix*, Table S2). Further analysis of the nucleotide binding site showed that the open RecA domain conformation is incompatible with ADP binding by Prp43 (Fig. 2*A*). While ADP is bound through interactions with both RecA domains in the closed state structure, only the interactions to one of the domains could be maintained when modeling ADP in the open state structures, suggesting that ADP is released when Prp43 enters the O state. To test this idea, we measured the dissociation rate of *N*-methylanthraniloyl-ADP (mant-ADP) from Prp43 by a stopped-flow setup (Fig. 2*B*). In the absence of Pfa1(gp) and ssRNA, mant-ADP release followed a single exponential time course with a slow rate constant of k_off_ = 0.002 s^−1^. Mant-ADP release was accelerated by threefold in the presence of ssRNA and by 100-fold in the presence of Pfa1(gp) (Fig. 2*B*), indicating that the opening of RecA domains observed by smFRET correlates with fast ADP release rates (Fig. 1 *D* and *G*). In further agreement with the smFRET data, the dissociation of mant-ADP was even faster in the presence of both Pfa1(gp) and ssRNA (*SI Appendix*, Fig. S4*A*). The low affinity of Prp43 for mant-ADP under these conditions resulted in a very low signal amplitude (*SI Appendix*, Fig. S4*B*). Previous studies have shown that Prp43 and Prp43-gp complexes in the nucleotide-free state target their RNA substrates with higher affinity than in the ADP state (10, 41). The low-affinity state allows Prp43 to move along the RNA, presumably because of reduced contacts between RecA2 and the RNA (10). Hence, the fast ADP release induced by Pfa1(gp) accelerates the transition back to the tight RNA binding state and might prevent Prp43 dissociation during translocation.

### Pfa1(gp) Enhances RNA Binding of Prp43 and Enables Processive Unwinding of Duplex RNA Structures.

**Fig. 3.**
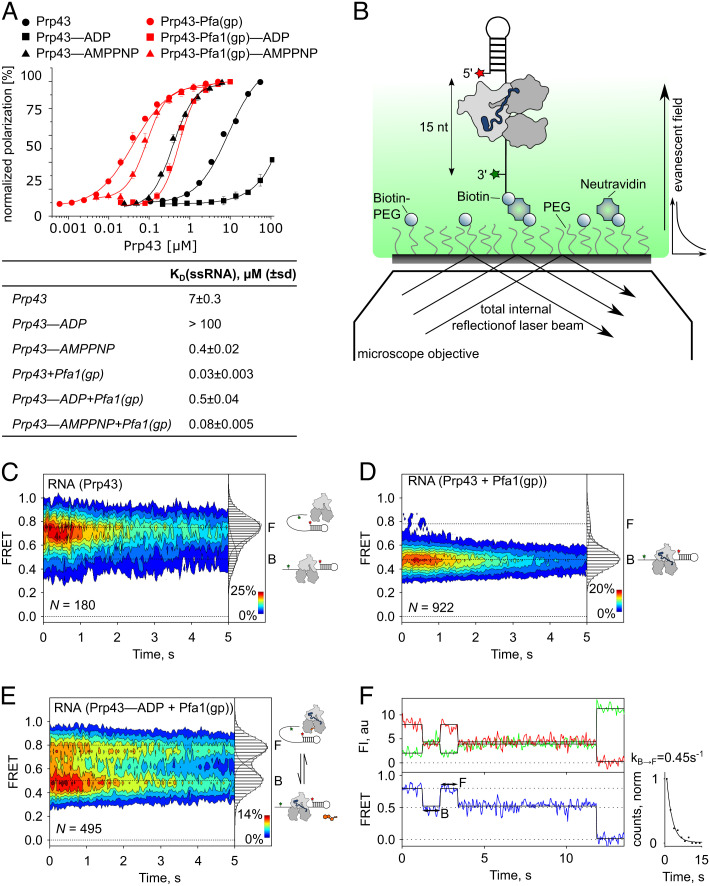
Pfa1(gp) enhances RNA binding of Prp43. (*A*) scPrp43 binding affinity to ssRNA determined by fluorescence polarization spectroscopy in the absence (black) or presence (red) of Pfa1(gp) in the nt-free (circles), ADP- (squares), or AMPPNP- (triangles) state. Shown are mean values; error bars correspond to the SD derived from N = 3 independent measurements. Normalization was performed by polarization values. The table indicates affinity constants (K_D_(ssRNA) ± SD). For Prp43–ADP less than 50% polarization was reached, indicated K_D_ is an estimate of the lower limit. (*B*) TIRF microscopy experiment scheme to monitor single-strand RNA binding and duplex unwinding by Prp43. The 3′-end of the RNA probe is biotinylated for attachment on neutravidin-functionalized coated coverslips. Distance changes between Cy3 (position 76, red star) and Cy5 (position 1, green star) report on conformational changes of the probe related to the binding of Prp43 and the unwinding of the stem loop. (*C*–*E*) Contour plots and 2D histograms showing the distribution of FRET values (mean ± SD, derived from N = 3 independent data sets) of the RNA probe in the presence of (*C*) Prp43(apo) (0.79 ± 0.01 and 0.51 ± 0.02), (*D*) Prp43-Pfa1(gp) (0.78 ± 0.03 and 0.48 ± 0.02), or (*E*) Prp43–ADP-Pfa1(gp) (0.80 ± 0.02 and 0.51 ± 0.02). Cartoons indicate the state of the RNA (free (F) or bound (B) by Prp43). (*F*) Representative time traces of Cy3- (green) and Cy5- (red) fluorescence intensity (FI) and FRET (blue, *Bottom* plot) corresponding to transitions between the B and the F state. Solid lines represent the Hidden–Markov fit of the traces. The distribution of dwell times of the B state, normalized by the number of FRET counts, was fitted by an exponential function to determine the dissociation rate of Prp43 from the RNA (k_B→F _= 0.45 s^−1^). n = 76 transitions were included in the analysis.

To further investigate how Pfa1(gp) modulates Prp43 interaction with the RNA substrate, we determined the binding affinity in our system (Fig. 3*A* and *SI Appendix*, Fig. S5). Consistent with previous results (10, 34, 41), the affinity of Prp43 for ssRNA in the absence of nucleotide was in the µM range without Pfa1(gp) and in the nM range in the presence of Pfa1(gp) (K_D _= 7 µM and K_D _= 0.03 µM, respectively, Fig. 3*A*). In the presence of Adenylyl-imidodiphosphate (AMPPNP), the affinity of Prp43 was slightly increased, while the affinity of the Prp43–Pfa(gp) complex was similar compared to the affinity of the complex in the nucleotide-free states (K_D_ = 0.4 µM and K_D_ = 0.08 µM, respectively). In the presence of ADP, however, the affinity decreases severely without Pfa1(gp), the K_D_ was so high that a full titration was not feasible (K_D_ > 100 µM). The Prp43-Pfa1(gp) complex showed moderate affinity in the presence of ADP (K_D_ = 0.5 µM), confirming that the G-patch promotes the binding of the helicase to the substrate and that the choice of the nucleotide (ADP, AMPPNP, or nt-free) regulates the switch between weak or strong binding states.

Next, we visualized time-resolved RNA binding by Prp43. We designed an RNA probe with a single-strand 3′-overhang allowing for Prp43 binding, followed by a stem loop, which is separated during Prp43 translocation (Fig. 3*B*). The 3′-end of the substrate was biotinylated to immobilize the probe on the cover slip, the Cy3-dye was placed in the single-strand region (position 76), and the Cy5-dye was placed at the 5′-end (position 1) such that the distance between the dyes is 15 nucleotides. In the absence of the helicase, the probe shows stable FRET signals with mean values of E_FRET _= 0.8 (*SI Appendix*, Fig. S6 *A* and *B*). After incubation with Prp43(apo), the majority of RNA molecules (76%) showed still stable signals with E_FRET_=0.79, similar to the free (F) RNA probe, and a smaller fraction (24%) displayed signals with E_FRET _= 0.51, corresponding to RNA bound (B) by the helicase (Fig. 3*C* and *SI Appendix*, Fig. S6*A* and Table S3). The addition of ADP shifted the distribution of FRET values entirely to the F state, which agrees with the very low binding affinities observed by fluorescence polarization (Fig. 3*A* and *SI Appendix*, Fig. S6*C*). In the presence of AMPPNP, the distribution is shifted slightly toward the B state, which is also consistent with the fluorescence polarization data (Fig. 3*A* and *SI Appendix*, Fig. S6*D*). Binding of the Prp43–Pfa1(gp) complex shifted the population toward the B state (88%), while high concentrations of Pfa1(gp) without Prp43 resulted only in the F state, confirming that the G-patch enhances Prp43 binding to the RNA (Fig. 3*D* and *SI Appendix*, Fig. S6*E* and Table S3). Upon the addition of ADP, the probe interconverted between F and B states indicating binding and dissociation of the Prp43–Pfa1(gp) complex (Fig. 3*E* and *SI Appendix*, Table S3). Traces with B to F transitions were used to estimate the dissociation rate of the complex in the low-affinity ADP state (k_off _= 0.45 s^−1^, Fig. 3*F* and *SI Appendix*, Table S4). This rate is one order of magnitude lower compared to the rate of ATP turnover (k_cat _= 5 s^−1^, *SI Appendix*, Fig. S1*B*). Hence, binding, hydrolysis, and release of ATP are much faster than dissociation of the complex from the RNA in the weak-affinity state, suggesting that Prp43-Pfa1(gp) can perform multiple rounds of ATP turnover while bound to the substrate.

**Fig. 4.**
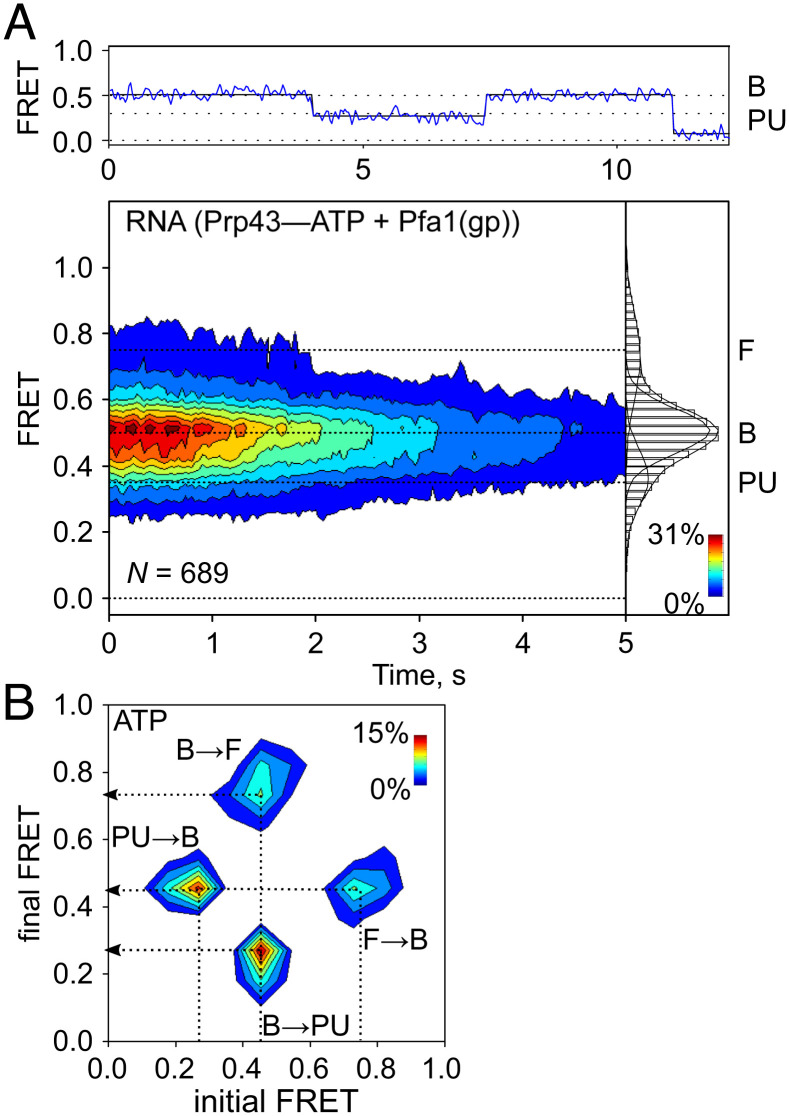
Duplex RNA unwinding by Prp43–Pfa1(gp) complex upon ATP binding. (*A*) Representative FRET trace (*Above*), contour plot, and 2D histogram (*Below*) showing the distribution of FRET values (mean ± SD, derived from N = 3 independent data sets) of the RNA probe in the presence of the Prp43–Pfa1(gp) complex at 2 µM ATP (0.76 ± 0.02 (F), 0.51 ± 0.02 (B) and 0.35 ± 0.05 (partially unwound, PU)). (*B*) Transition density plot visualizing the frequency of transitions between B and F (B→F, F→B) or B and PU (B→PU, PU→B) states.

Next, we investigated the interaction between the RNA and Prp43-Pfa1(gp) in the presence of the physiological substrate ATP (Fig. 4). At high ATP concentrations (2 mM), FRET signals vanish immediately because rapid unwinding of the stem loop increases the Cy3-Cy5-distance to 75 nt, which is far beyond the FRET range. At very low ATP concentration (2 µM), where the binding rate of the nucleotide limits the turnover of the helicase, the majority of molecules is in the B state. However, a fraction of the population shows a new state with E_FRET_=0.35 (Fig. 4*A* and *SI Appendix*, Table S3). We assume that Prp43 binding straightens the RNA probe leading to the decrease in FRET in the B state. Hence, further reduction of FRET values most likely corresponds to the partial unwinding (PU) of the stem loop. In 20% of the duplex RNA molecules, the PU state is transiently sampled (Fig. 4*B*). The analysis of the transition state frequency between B, F, and PU states revealed that 33% of observed transitions occur between B and PU or PU and B, indicating that a significant portion of RNA molecules undergo partial unwinding (Fig. 4*B*). A very small number of molecules showed direct transitions between F and PU, presumably the B state was too short-lived to be captured in our experiment. To distinguish events resulting from ATP binding or the hydrolysis of ATP, we repeated the experiment using AMPPNP, which is hydrolyzed at a very low rate by Prp43 (*SI Appendix*, Fig S7*A*). However, the distribution of FRET states of the RNA probe, in particular the sampling of the PU state, was similar as in the experiments with ATP (*SI Appendix*, Fig S7*B*). The PU state was also sampled when we incubated the probe with a Prp43 variant (Prp43_E216A_) that hydrolyses ATP at very low rate, comparable to the AMPPNP hydrolysis rate by Prp43(wt) (*SI Appendix*, Fig. S7 *A* and *C*) (8, 44). In the time course of our experiments (33 s), not more than one nucleotide is hydrolyzed either by Prp43-Pfa1(gp)–AMPPNP or Prp43_E216A_-Pfa1(gp)–ATP (k_cat_ = 0.04 s^−1^, *SI Appendix*, Fig. S7*A*), indicating that the binding and potentially hydrolysis of a single ATP are sufficient to induce partial unwinding of the RNA probe, i.e., the transition from the B to the PU state. A previously published translocation model derived from structural analysis of the DEAH-box helicase DHX37 suggested that the power stroke for translocation occurs during ATP binding, rather than during hydrolysis (9). This model would explain why our RNA probe is unwound in an ATP-dependent manner but also in the absence of continuous ATP hydrolysis. The translocation model further proposes that when DEAH-box helicases approach dsRNA, the conformational changes induced by ATP binding separate dsRNA by a winching-like mechanism (9, 10, 19). The difference in the FRET efficiencies of the B and the PU state suggests that the Cy3- and Cy5-fluorophores in our RNA probe reorient by 0.8 nm (45, 46), which would correspond to the separation of 2–3 base pairs considering a rise per base pairs of 0.3 nm in an RNA double helix (47). Therefore, at least for the first unwinding step of the RNA duplex, the binding and potentially the hydrolysis of a single ATP is sufficient for the melting of several base pairs.

**Fig. 5.**
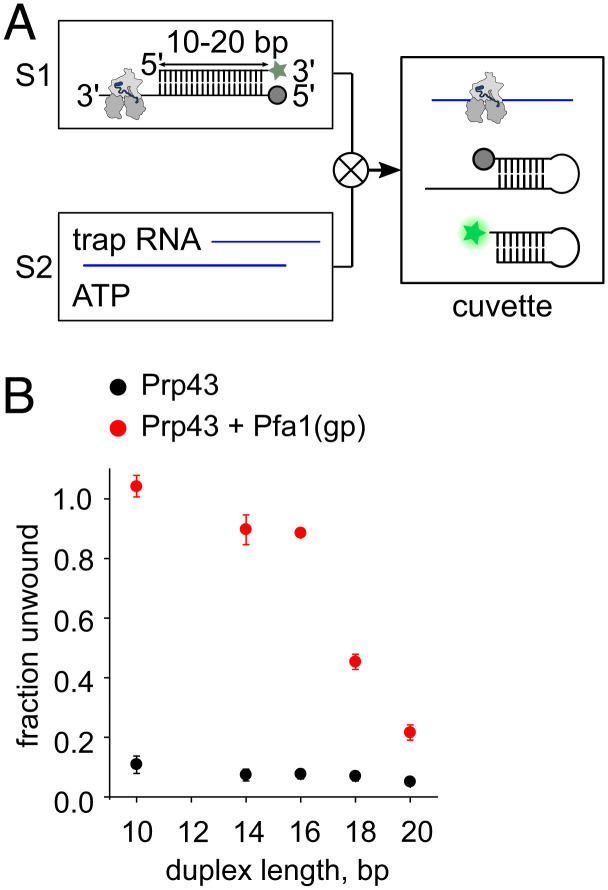
Separation of long RNA duplexes by Prp43–Pfa1(gp) complex. (*A*) Stopped-flow experiment scheme monitoring unwinding of duplex RNA. Syringe 1 (S1) contains duplex RNA constructs labeled by ATTO488 (green star) and Eclipse quencher (grey circle) preincubated with Prp43 or Prp43–Pfa1(gp) complex. Syringe 2 (S2) contains Poly U RNA (100 µg/ml) and 4 mM ATP. Upon rapid mixing, the separation of duplex strands becomes apparent by dequenching of the ATTO488 fluorophore (bright green star). Single strands fold into stem loops to prevent reannealing. (*B*) Fraction of duplex RNA unwound by Prp43 in the presence (red) and absence (black) of Pfa1(gp), depending on duplex length. The maximum amplitude of the normalized fluorescence time traces was used to indicate the fraction of unwound RNA. Shown are mean values; error bars correspond to the SD derived from N = 3 independent measurements.

Further unwinding leads to the increase of the Cy3- and Cy5-distance in the RNA probe beyond the limits for FRET detection. To address, whether Prp43 can separate longer RNA duplexes in successive translocation steps on the same RNA substrate during continuous ATP consumption we followed a rapid kinetics approach (Fig. 5*A*). We designed complementary RNA strands of 10, 14, 16, 18, and 20 base pair length with an ATTO488 fluorescence label at the 3′-end of the top strand and an Eclipse quencher at the 5′-end of the bottom strand. The bottom strand contains a 12 nt single-strand region at the 3′ end, accommodating a single Prp43 molecule (Fig. 5*A*). We preformed Prp43–RNA and Prp43–Pfa1(gp)–RNA complexes in the absence of ATP. Using stopped-flow setup, we rapidly mixed them with high concentrations of ATP and single-stranded PolyU RNA. The unwinding of each duplex is caused by the action of a single helicase as rebinding of Prp43 after dissociation from the duplex is prevented by the binding to the trap RNA (PolyU). Separated strands fold into a stem loop structure in order to avoid reannealing. We monitor the time course of RNA unwinding by the dequenching of ATTO488 fluorescence during strand separation. Signal changes corresponding to RNA unwinding by the Prp43–Pfa1(gp) complex show a complex fluorescence signature comprising a lag-phase, steep fluorescence increase and a fluorescence plateau, which then slowly reaches stable fluorescence end levels indicative of a complex multistep kinetic process (*SI Appendix*, Fig. S8*A*). To interpret the stopped-flow signals with respect to the unwinding of the RNA constructs, we therefore normalized the data by division by the fluorescence amplitude corresponding to the complete unwinding of the RNA duplexes (*SI Appendix*, Fig. S8*B*). The Prp43–Pfa1(gp) complex separates the 10, 14, and 16 bp RNA constructs completely (89 to 100% unwinding, Fig. 5*B*). The 18 and 20 bp constructs also show significant unwinding (maximum amplitudes are 45% and 22%, respectively, Fig. 5*B*). After reaching the maximum fluorescence level, traces corresponding to the 18 bp and 20 bp construct decrease to stable fluorescence end levels (*SI Appendix*, Fig. S8*B*). This is presumably caused by a partial reannealing of separated strands that competes with the stem loop formation. Next, we manually dissected the lag phase preceding the actual strand separation, and analyzed the duration with respect to the duplex length (*SI Appendix*, Fig. S8*C*). As the dequenching of the ATTO488 dye only occurs when the Prp43–Pfa1(gp) complex reaches the 5′ end of the bottom strand, the lag-phase correlates in first approximation with the translocation time. The Prp43–Pfa1(gp) complex requires significantly more time to arrive at the 5′-end of longer duplexes, which is a strong indicator for an unwinding process that involves multiple successive translocation steps on the same RNA (*SI Appendix,* Fig. S8*C*). It appears that the longest duplex (20 bp) is close to the maximum range of unwinding by the Prp43–Pfa1(gp) complex. This range exceeds the size of the first unwinding step significantly (2–3 bp, Fig. 4*A*), providing further evidence that Prp43 performs several catalytic cycles without detachment. We observe almost no unwinding of RNA duplexes by Prp43 in the absence of Pfa1(gp), indicating that individual Prp43 molecules are incapable of continuous unwinding (*SI Appendix*, Fig. S8 *A* and *B* and Fig. 5*B*). Therefore, the interaction with G-patch partners enables Prp43 to separate duplex strands in a processive manner.

### Pfa1(gp) Accelerates Transitions between Closed and Open RecA Domain Conformation during ATP Turnover, Facilitating Processive Translocation.

**Fig. 6.**
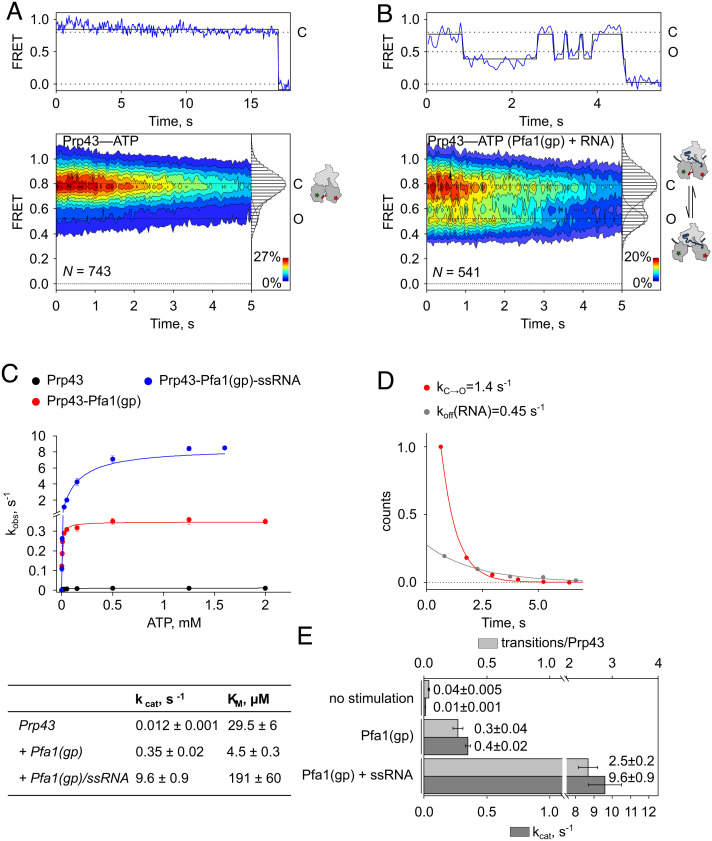
Pfa1(gp) and ssRNA accelerate conformational cycling of RecA domains during ATP turnover. (*A* and *B*) Representative FRET trace (*Above*), contour plots and 2D histograms (*Below*) showing the distribution of FRET values (mean ± SD, derived from N = 3 independent data sets) of (*A*) Cy3-/Cy5-labeld Prp43–ATP (0.80 ± 0.02 and 0.53 ± 0.02) or (*B*) Cy3-/Cy5-labeld Prp43–ATP in complex with Pfa1(gp) and in the presence of ssRNA (0.80 ± 0.01 and 0.52 ± 0.01). (*C*) Michaelis–Menten titration and table with ATP turnover numbers (k_cat_) and dissociation constants (K_M_) showing the stimulation of Prp43 ATPase activity by Pfa1(gp) and ssRNA. (*D*) Rates of RecA domain opening (k_C→O_, red) and the RNA dissociation rate (k_off_(RNA), grey) of the Prp43–Pfa1(gp) complex on ssRNA. (*E*) Comparison between transitions per molecule Prp43 as observed in the smFRET experiment (light grey) and the ATPase turnover numbers (k_cat_) determined in *C* (dark grey). The transitions per molecule Prp43 were determined by dividing the number of transitions between C and O state by the number of individual traces (*N*). In the case of stimulation by Pfa1(gp) and ssRNA only detectably activated molecules, i.e., traces that showed at least one transition, were included.

Knowing that continuous turnover is necessary for Prp43 to unwind more than the initial base pairs of an RNA duplex, we investigated the conformational dynamics of Cy3-/Cy5-labeled Prp43 at high ATP concentrations (Fig. 6). In the absence of Pfa1(gp) or ssRNA, we mainly observed stable signals corresponding to the C state, which were earlier observed for Prp43(apo) and Prp43–ADP (Figs. 1 *C* and *E* and 6*A* and *SI Appendix*, Table S1). However, a small part of the population (13%) showed the O state, which was so far only observed in the presence of Pfa1(gp) or ssRNA (Fig. 1 *D* and *F*–*H*). Few traces (4%) showed transitions between C and O states. Considering that domain opening occurs concomitantly to ADP release (Fig. 2) and domain closures results from nucleotide binding, slow dynamics is expected given that the intrinsic ATP hydrolysis rate of the helicase is very low (k_cat _= 0.01 s^−1^, Fig. 5*C*). In the presence of AMPPNP, the RecA domains exclusively showed the C state, indicating that the slow dynamics is indeed a result of ATP hydrolysis (*SI Appendix*, Fig. S9 and Table S1). To examine the influence of Pfa1(gp) and ssRNA on the motility of the RecA domains, we first monitored smFRET of the Prp43–Pfa1(gp) complex in the absence of ssRNA. The complex showed increased levels of the O state (35%) and 27% of molecules toggled between C and O, which is a significant increase compared to unstimulated Prp43 and agrees with elevated rates of ATP turnover (k_cat _= 0.4 s^−1^, Fig. 6*C* and *SI Appendix*, Fig. S10 *A* and *B* and Table S3). The incubation of the Prp43–Pfa1(gp) complex with ssRNA further stimulated the rate of ATP hydrolysis (k_cat _= 9.6 s^−1^, Fig. 6*C*). The distribution of states did not change, but the activation of Prp43 increased further, which becomes apparent by higher exchange rates between C and O states (k_C→O _= 1.44 s^−1^, k_O→C _= 1.92 s^−1^, Fig. 6*B* and *SI Appendix*, Table S4). The conformational distribution and exchange rates were independent from the label positions as the control mutant Prp43_Cys_2 with a different label position in RecA1 showed similar transitions rates (*SI Appendix*, Fig. S11 and Table S4). The rate of ATP turnover determined in steady-state kinetic experiments is somewhat faster than the rate of conformational transitions observed by smFRET, presumably due to reduction of activity caused by the immobilization process. Still, k_C→O_ is faster than the previously determined dissociation rate from the RNA (k_off _= 0.45 s^−1^, Fig. 3 *E* and *F* and *SI Appendix*, Table S4), indicating that the helicases convert fast enough from the weak (ADP) to the strong (nt-free) RNA binding state to complete several conformational cycles before detachment.

## Discussion

### Motility Cycle of the Prp43–Pfa1(gp) Complex.

**Fig. 7.**
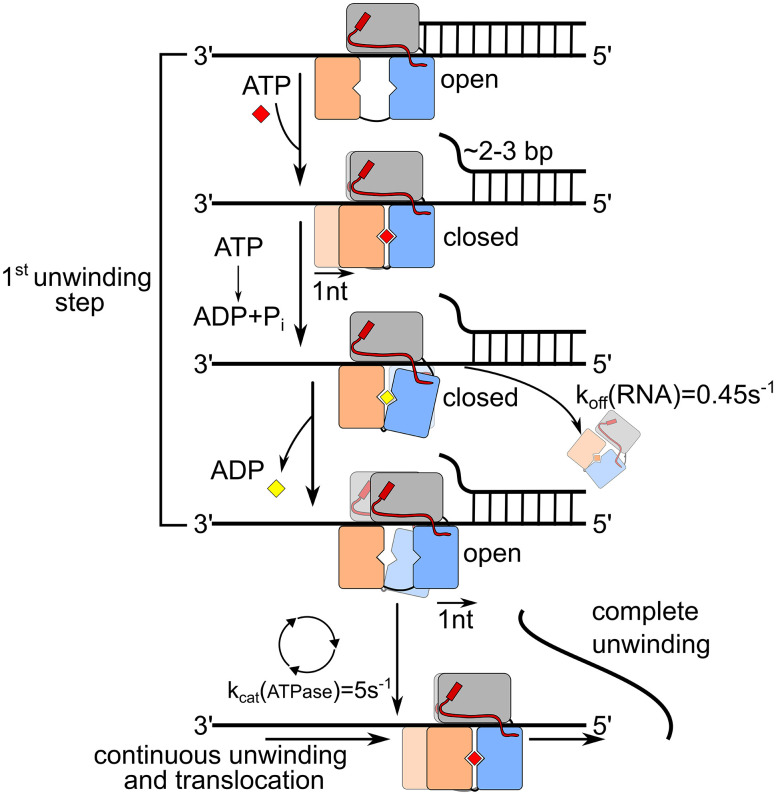
Model of the motility cycle of the Prp43–Pfa1(gp) complex. Pfa1(gp) is depicted in red, the C-terminal domains of Prp43 are colored in grey, and RecA1 and RecA2 are shown in orange and blue, respectively. The model describes how the ATP-dependent conformational cycling of the Prp43–Pfa1(gp) complex induces weak and strong RNA binding states leading to processive translocation and unwinding of RNA. Prp43 affinity constants for ssRNA are derived from fluorescence polarization experiments (Fig. 3*A*), the Prp43 drop-off rate corresponds to k_B→F_ of the RNA probe (Fig. 3 *E* and *F*) and the ATP turnover rate was determined by Michaelis–Menten titration (*SI Appendix*, Fig. S9).

The DEAH/RHA helicase Prp43 samples both open and closed RecA domain conformations. In the absence of RNA and G-patch factor, RecA domains rarely enter the O state such that nucleotide release and ATP turnover are slow. Without G-patch, the affinity of Prp43 for RNA is low and the dissociation from the RNA occurs with higher probability than the translocation in 5′ direction. Overall, Prp43 is in a state where continuous unwinding of structured RNAs is hardly possible. Our data show that Pfa1(gp) promotes transitions between open and closed conformations of RecA domains during ATP turnover resulting in processive translocation and unwinding of duplex RNA (Fig. 7). In the nucleotide-free state, the Prp43–Pfa1(gp) complex strongly binds ssRNA, ATP binding leads to closure of the RecA domains. During the transition to the closed state, Prp43 translocates on the RNA separating duplex strands. Structural analysis of Prp43 and other DEAH/RHA helicases has shown that translocation occurs in steps of one nucleotide per hydrolyzed ATP (9, 10). In our study, domain closure and concomitant translocation can separate the initial 2–3 bp of a duplex strand, suggesting that the step size of translocation might differ from the step size of RNA unwinding. It remains to be established whether subsequent unwinding steps occur also in increments of several nucleotides. The hydrolysis of ATP induces the opening of the RecA domains. While ADP is still bound, the affinity to the RNA is low, because RecA2 has little contact with the substrate (10) and the helicase is susceptible to dissociation. Pfa1(gp) accelerates domain opening and ADP release, thus promoting the transition back to tight RNA binding. Assuming that the conformational transitions are strictly coupled to the hydrolysis of ATP, the ATP turnover rate (k_cat_(scPrp43) = 5 s^−1^, *SI Appendix*, Fig. S12) corresponds to the rate of translocation (5 nt/s). The dissociation of the Prp43–Pfa1(gp) complex is much slower (k_off_(scPrp43) = 0.45 s^−1^), indicating that the helicase undergoes several catalytic cycles before dissociation from the RNA. Continuous turnover enables the Prp43–Pfa1(gp) complex to separate duplex strands of up to 20 bp. In our experiments, strand separation takes only place when Prp43 translocates across the entire duplex, which would require 20 processive translocation cycles.

### Strand Separation in the Cellular Context.

RNA unwinding by Prp43 relies on continuous conformational cycling of the RecA domains. Most likely, the transition to the closed state during ATP binding leads to the separation of proximal duplex strands. The underlying principle resembles the winching mechanism applied by the spliceosomal helicases Prp16, which remodels the spliceosomal C complex after the first splicing reaction to enable exon ligation, and Prp22, which subsequently releases the mature mRNA from the complex (48–51). Both helicases change the structure of their target RNAs inside the spliceosome without direct contact, supposedly by pulling on the RNA thereby remodeling the RNP structure (19). As part of the ILS, Prp43 binds to the 3′-end of U6 snRNA disrupting its interactions with U2 snRNA and the intron, which triggers spliceosomal disassembly (52). The location of the helicase binding site is distant from the U6 snRNA-intron duplex, indicating that Prp43 can transmit force over long distances similar to Prp16 or Prp22. Together, these observations suggest that DEAH/RHA helicases destabilize RNA structures when direct access to the target site is restricted (53, 54). The landing zone of Prp43 in the ILS consists of not more than 15 nucleotides (52). Hence, processive translocation along approximately 20 nucleotides is sufficient for the helicase to reach the physical barrier of the RNP and disrupt its RNA structure. However, compared to other structurally related NS3 and NPH-II helicases, which unwind duplexes structures of up to 40 bp, the range of processive unwinding by Prp43 is relatively small (55, 56).

### Motility and Strand Separation by DEAD-Box Helicases.

RNA unwinding by Prp43 is clearly distinct from the mechanism employed by the structurally related DEAD-box helicases, which unwind structured RNAs by local strand displacement in a nonprocessive manner (57). Without ligands, RecA domains of DEAD-box proteins are open and close upon the joint binding of ATP and RNA forcing it in a bent conformation incompatible with duplex structure (58–67). Upon ATP hydrolysis and presumably after phosphate release the RecA domains return to the open conformation, releasing the remaining RNA strand and allowing the enzyme to move on to the next substrate (62, 68). Analogous to our observations on Prp43, ATP binding initiates the power stroke required for strand displacement. However, DEAD-box helicases do not translocate along the RNA, and therefore, only local structures can be unwound. Some DEAD-box helicases perform processive unwinding of the same duplex in complex with auxiliary factors. An example is eIF4A, which unwinds the structured 5′ untranslated regions (5′UTRs) of mRNAs synergistically with components of the m7-G-cap-binding complexes eIF4F, in particular, eIF4G, eIF4B, and eIF4H (69–73). eIF4G stabilizes the half-open RecA domain conformation in eIF4A, while eIF4B and eIF4H improve the overall affinity toward RNA, preventing together the dissociation from the substrate (66, 73). Conformational guidance by cofactors stabilizing structures with high substrate affinity seems to be a common theme modulating the processivity of both types of helicases.

### Regulation of DEAH/RHA Helicases by Cofactors.

Cofactors play a central role in the regulation of DEAH/RHA helicases, which have low intrinsic substrate specificity and therefore require activation in a specific cellular environment avoiding futile ATP hydrolysis and undesired RNA unwinding. Prp2/DHX16 is a further DEAH/RHA family member known to interact with a G-patch partner (74–77). In yeast spliceosomes, Spp2 tethers Prp2 to adjacent spliceosomal proteins where it facilitates rearrangements making the branch point accessible for the splicing reaction (74, 75, 78–80). Although the Spp2 gp motif braces the RecA2 and WH domain of Prp2 in a similar manner as observed in the DHX15–NKRF(gp) complex, Spp2 does not alter the conformation of Prp2 and is not capable to stimulate the ATPase activity of Prp2 independently (40, 41, 78, 81). The activation occurs exclusively in the presence of RNA, suggesting that the regulation of Prp2 follows different principles than Prp43. Presumably tethering of Prp2 to the single-stranded intron region of the pre-mRNA is more relevant than the stabilization of an open RecA domain conformation. Cellular targets of the many G-patch factors identified in the human proteome remain to be identified (25).

We demonstrate how conformational cycling of a DEAH/RHA helicase correlates with processive translocation along the RNA substrate. Remaining open questions concern how translocation is regulated in the cellular context, i.e., as part of spliceosome or in nascent ribosome complexes. In vivo, different G-patch factors compete for the interaction with Prp43 (34) and might affect the speed of translocation or the degree of processivity to different extents. Knowledge on the conformational dynamics of other DEAH/RHA helicases will provide important insights into the regulation of fundamental cellular processes.

## Material and Methods

Detailed descriptions of the experimental procedures are provided in *SI Appendix*, *Materials and Methods**.*

All Prp43 and Pfa1 constructs were expressed in *Escherichia coli* Rosetta II and first purified by glutathione sepharose affinity chromatography using the N-terminal glutathione S-transferase (GST)-tag. Prp43 constructs were then subjected to affinity chromatography using their C-terminal His_6_- or Strep-tag. Finally, all Prp43 and Pfa1 constructs were purified to homogeneity by size exclusion chromatography.

Prp43 was fluorescently labeled with a mixture of Cy3-maleimeide and Cy5-maleimide using reactive cysteines at position C303 and K170C. The labeled protein was separated from excess dye by Ni-sepharose affinity chromatography.

smFRET experiments were performed using a commercial TIRF microscopy setup (Olympus, Japan) described in ref. 42. Polyethylen glycole (PEG) -Biotin functionalization of cover slips was performed as described in ref. 42. The concentration of Cy3/Cy5-labeled ctPrp was 1 nM. ctPrp43–Pfa1(gp) complex was formed prior to immobilization incubating 1 µM labeled ctPrp43 with 5 µM ctPfa1(gp). The concentration of the labeled RNA construct was 1 nM. TIRF microscopy buffers, single molecule detection, and data analysis were adapted from protocols described in ref. 42.

ATP turnover by Prp43 was monitored using a coupled enzymatic assay following nicotinamide adenine dinucleotide absorption. The binding of Pfa1(gp) to Prp43 was monitored by isothermal titration calorimetry (ITC) with a MicroCal VP-ITC (Malvern Panalytical), and the RNA binding of Prp43 was measured by fluorescence polarization spectroscopy using a VICTOR Nivo Multimode Microplate Reader (PerkinElmer). ADP release from Prp43 was followed through FRET between Prp43’s tryptophan residues and mant-labeled ADP using a stopped-flow apparatus (Applied Photophysics). Using the same stopped-flow apparatus, unwinding of RNA duplex constructs was monitored by the dequenching of ATTO488 fluorescence upon distance increase to the Eclipse quencher attached the 5′- and 3′-ends of partner strands.

## Supplementary Material

Appendix 01 (PDF)

## Data Availability

All study data are included in the article and/or *SI Appendix*.
