# Supplementary material for: Regulation of the DEAH/RHA helicase Prp43 by the G-patch factor Pfa1
Source: Proc Natl Acad Sci U S A. 2022 Nov 21;119(48):e2203567119. doi: 10.1073/pnas.2203567119 (PMC9860317; doi:10.1073/pnas.2203567119)
Supplement: Supplementary file 1 — Appendix 01 (PDF) [file pnas.2203567119.sapp.pdf]

PNAS
www.pnas.org

Supplementary Information for

Regulation of the DEAH/RHA helicase Prp43 by the G-patch factor Pfa1

Marieke Enders[1], Ralf Ficner[1], Sarah Adio[1]*

Department of Molecular Structural Biology, Institute of Microbiology and Genetics, Georg-August-University Göttingen, Justus-von-Liebig-Weg 11, D-37077 Göttingen, Germany

*Sarah Adio

Email: sarah.adio@uni-goettingen.de

This PDF file includes:

Supplementary Information Text

Figures S1 to S12

Tables S1 to S4

SI References

Supplementary Information text

Materials and Methods

Protein expression and purification

The homologues of Prp43 and Pfa1 from *C. thermophilum* (ctPrp43, ctPfa1) are annotated as 'hypothetical protein CTHT_0005780' and 'hypothetical protein CTHT_0048220'. Full-length ctPrp43 and the gp motif of ctPfa1 (residues 662-742) were cloned from genomic DNA of *C. thermophilum var. thermophilum* DSM 1495 into pGEX-6P-1 (1). For ctPrp43, a C-terminal non-cleavable His_6-tag was added to the GST-fusion protein by site-directed mutagenesis. *S. cerevisiae* Pfa1(gp) (residues 701-767) was cloned into pGEX-6P-1 (2). Prp43 from *S. cerevisiae* was amplified from genomic DNA of *S. cerevisiae S288c* using primers CCGTCGACATGGGTTCCAAAAGAAGATTC and GGGCGGCCGCCTA TTTCTTGGAGTGCTTAC and cloned into pGEX-6P-1 using the SalI and NotI restriction sites. All dispensable bases between the PreScission Protease cleavage site and the starting methionine of scPrp43 were deleted and a Strep-tag was added at the C-terminus of the GST-fusion protein *by* site-directed mutagenesis.

scPrp43, ctPrp43 and ctPfa(gp) constructs were recombinantly expressed in Rosetta 2 (DE3) cells using an autoinduction protocol adapted from Studier (3). Expression cultures were grown in ZY medium, supplemented with 25 mM K_2HPO_4, 25 mM NaH_2PO_4, 50 mM NH_4Cl, 5 mM Na_2SO_4, 0.5 % (v/v) glycerol, 0.05 % (w/v) glucose, 0.2 % (w/v) α-lactose, 2 mM $MgSO_4$ and a mixture of trace metals (3) at 37°C at 220 rpm until an OD600 of 0.4 was reached. Then, the temperature was reduced to 16°C and the cultures were incubated at 220 rpm for ~60h.

scPfa1(gp) was recombinantly expressed in BL21 (DE3) cells. Expression cultures were grown in 2YT medium at 37°C at 220 rpm until an OD600 of 0.8 was reached. The expression was induced by adding isopropyl β-D-1-thiogalactopyranoside (IPTG) to a final concentration of 0.5 mM, and the cultures were incubated at 16°C and 220 rpm overnight.

Harvested cells were disrupted using a microfluidizer (Microfluidics) in 50 mM Tris/HCl (pH 7.5), 500 mM NaCl, 5% (v/v) glycerol and 10 mM ethylenediaminetetraacetic acid (EDTA), soluble proteins were isolated by ultracentrifugation at 35000x*g* for 30min. The clarified lysate was loaded onto Glutathione Sepharose 4B (Cytiva) at 20°C. Bound nucleic acids were removed by intensive washing with lysate buffer supplemented with 2 M LiCl and the target protein was eluted with 30 mM reduced glutathione. The GST-tag was proteolytically cleaved off with 1:100 (w/w) PreScission protease overnight at 4°C. After cleavage, two additional amino acids (Gly-Pro) remain at the N-terminus of the proteins. Strep-tagged Prp43 was loaded onto StrepTactin HP Sepharose (GE Healthcare) in 50 mM Tris/HCl (pH 7.5), 400 mM NaCl, 5% (v/v) glycerol, 2 mM $MgCl_2$, and ctPrp43-His was loaded onto Ni Sepharose HP (GE Healthcare) in 50 mM Tris/HCl (pH 7.5), 400 mM NaCl, 5% (v/v) glycerol, 2 mM $MgCl_2$ and 10 mM imidazole. Elution of target protein was realized with 3 mM D-desthiobiotin (scPrp43-Strep) or 500 mM imidazole (ctPrp43-His). All proteins were purified to homogeneity by size exclusion chromatography (Superdex 200 or Superdex 75, Cytiva for Prp43 and Pfa1 respectively) in 20 mM Tris/HCl (pH 7.5), 200 mM NaCl, 5% (v/v) glycerol, 2 mM $MgCl_2$. For the gp constructs, the buffer contained 400 mM NaCl. The proteins were concentrated to a final concentration of 70-300 µM (Prp43; Amicon Ultra 50K, Millipore) or 150-700 µM (Pfa1(gp); Amicon Ultra 3K, Millipore). scPrp43-E216A was expressed and purified using the same protocol as described for scPrp43. Protein purity was assessed by SDS-PAGE. To adequately separate Prp43 and Pfa1(gp) variants, the gels contained 10% and

17.5% acrylamide respectively. As reference, a protein ladder ranging from 10 to 180 kDa (PageRuler Prestained Protein Ladder, Thermo) was used.

Fluorescence-Labeling of ctPrp43

To generate two maleimide-reactive fluorescence-labeling sites in ctPrp43, eight native cysteine residues had to be considered. C148, C214, and C377 are buried inside the protein and inaccessible to the coupling group. C303, C323, C441, C508, and C543 are surface exposed and therefore accessible to the fluorescence dye. C303 is located in the RecA2 domain and remained unchanged while the other exposed cysteines were replaced by site directed mutagenesis (C323V, C441A, C508A and C543S). The second labeling site was generated by introducing a cysteine residue at position K170 in the RecA1 domain. The resulting variant is denominated $Prp43_{Cys}$. A control mutant denominated $Prp43_{Cys}2$ was generated, where the labeling site in RecA1 was introduced at position S187. The mutant proteins were expressed and purified as described above with the addition of 5 mM DTT to the lysis buffer and 1 mM TCEP to the His-trap loading and elution buffers. The purified proteins were mixed with Cy3-maleimeide and Cy5-maleimide (Cytiva) dissolved in dimethylsulfoxide at a molar ratio of 1:2:3 (protein:Cy3:Cy5) and incubated for 10 min at 20°C. Excess dye was removed by Ni-sepharose affinity chromatography, labeled proteins were eluted in 50 mM Tris/HCl (pH 7.5), 400 mM NaCl, 5% (v/v) glycerol, 2 mM $MgCl_2$, 250 mM imidazole. The labeled proteins were dialyzed twice against 50 mM Tris-HCl, pH 7.5, 300 mM KCl, 3 mM $MgCl_2$ using Slide-A Lyzer Dialsis Casette G2 3.5K (Thermo) for 1 h at 4°C. The labeled proteins were concentrated to final concentrations between 40 and 70 µM (Amicon Ultra 50K, Millipore).

To determine the degree of labeling, i.e. the average number of fluorophore molecules per molecule Prp43, the absorption of the labeled protein was measured at 280 nm as well as at the absorption maxima of Cy3 and Cy5, 552 nm and 650 nm. Both Cy3 and Cy5 also show absorption at 280 nm, thereby increasing the A_{280} for the labeled protein. The correction factors (CF) required to eliminate the contribution of the dyes at 280 nm were provided by the manufacturer as CF_{Cy3}=0.08 and CF_{Cy5}=0.05 (Cytiva). The dye to protein ratio was then calculated for both dyes using

$$c(Prp43) = \frac{A_{280} - [CF_{Cy3} \cdot A_{552}] - [CF_{Cy5} \cdot A_{650}]}{\varepsilon_{280}},$$

$$D/_{P}\,(Cy3) = \frac{A_{552}/_{\varepsilon_{552}}}{c(Prp43)} \text{ and } D/_{P}\,(Cy5) = \frac{A_{650}/_{\varepsilon_{650}}}{c(Prp43)}$$

where A_{xxx} is the absorption of labeled Prp43 at the specified wavelength, ε_{280}=72700 $M^{-1}cm^{-1}$ is the extinction coefficient of ctPrp43 at 280 nm (ProtParam (4)), and ε_{552}=150000 $M^{-1}cm^{-1}$ and ε_{650}=250000 $M^{-1}cm^{-1}$ are the extinction coefficients of Cy3 and Cy5 at their absorption maxima. $Prp43_{Cys}$ was labeled by 77% with Cy3 and by 66% with Cy5. $Prp43_{Cys}2$ was labeled by 80% with Cy3 and by 81% with Cy5.

Sample preparation for TIRF microscopy

Cover slips and objective slides were cleaned by bath sonication in 1 M KOH and exposure to plasma (FEMTO plasma cleaner, Diener Electronic GmbH, Germany). Surfaces were then silanized by sonication in 3.9 mM N1-[3-(trimethoxysilyl) propyl] diethylenetriamine (Sigma-Aldrich) and 1.7 mM acetic acid, and baked for 20 min at 110 °C. PEG/PEG-Biotin functionalization of silanized surfaces was carried out by incubation with 20 mM PEG-NHS (MeO-PEG-NHS, IRIS Biotech GmbH, PEG1165), 0.2 mM Biotin-PEG-NHS (IRIS Biotech, PEG1057) and 20 mM KOH in 100 mM H_3BO_3 solution for 1 h at room temperature. Excess PEG was removed by washing with H_2O. Cover slips were dried at 60 °C and stored under vacuum. For TIRF experiments, flow chambers were generated by combining objective slides and cover slips with double-sided sticky tape.

The ctPrp43-ctPfa1(gp) complex was formed by incubating 1 µM labeled ctPrp43 with 5 µM ctPfa1(gp) in TIRF buffer A (50 mM Tris-HCl, pH 7.5, 300 mM KCl, 3 mM $MgCl_2$) for 10 min at room temperature. Prior to the experiment, the complex was diluted to 1 nM ctPrp43 with TIRF buffer supplemented with 5 µM ctPfa1(gp). For experiments without ctPfa1(gp), labeled ctPrp43 was diluted to 1 nM with TIRF buffer A. Biotin/PEG-functionalized cover slips were incubated for 5 min at room temperature with TIRF buffer A containing additionally 10 mg ml^{-1} BSA and 1 µM neutravidin (Thermo Scientific). Excess neutravidin was removed by washing the cover slip with the same buffer containing 1 mg ml^{-1} BSA. A biotinylated anti-His antibody (Rabbit monochlonal, Sigma) was applied at 1.25 µg/ml to the cover slip. Excess antibody was removed by washing with TIRF buffer A containing 1 mg ml^{-1} BSA. Labeled ctPrp43 or ctPrp43-ctPfa1(gp) complex (1nM final concentration) was applied to the surface and incubated for 1 min at room temperature. Images were recorded after washing with TIRF buffer C (TIRF buffer A with 2.5 mM protocatechuic acid, 50 nM protocatechuate-3,4-dioxygenase (from *Pseudomonas*), 1 mM trolox (6-hydroxy-2,5,7,8-tetramethylchromane-2-carboxylic acid) and 1 mM methylviologen). To study the influence of ssRNA and ADP, the imaging buffer was supplemented with 50 µg/ml PolyU RNA (Sigma Aldrich) or 100 µM ADP. To observe domain movement during ATP turnover, the imaging buffer was supplemented with 2 mM ATP, 0.1 mg/ml pyruvate kinase and 3 mM phosphoenolpyruvate.

The labeled RNA (G[Cy5]CGCCUACGCCACCAGCUCCGUAGGCGCAGGAGCGCCUACGGAGCU GGUGGCGUAGGCGCAAAAAAAAAAAAAAAU[Cy3]AAAAAAAAAAAAAAAAAAA-Biotin) was diluted to 1 nM with TIRF buffer B (50 mM Tris-HCl, pH 7.5, 150 mM KCl, 3 mM $MgCl_2$). Biotin-PEG-functionalized cover slips were incubated for 5 min at room temperature with TIRF buffer B containing additionally 10 mg ml^{-1} BSA and 1 µM neutravidin (Thermo Scientific). Excess neutravidin was removed by washing with the same buffer containing 1 mg ml^{-1} BSA. The labeled RNA was applied to the surface and images were recorded after the addition of TIRF buffer C. To observe binding of Prp43 to the RNA, the buffer was supplemented with 5 µM scPrp43 or 0.5 µM scPrp43 and 2.5 µM scPfa1(gp). The influence of nucleotides was studied by adding 2 mM ADP, AMPPNP or ATP with an energy recycling system (see above) to TIRF buffer C.

TIRF microscopy

TIRF imaging was performed on an IX 81 inverted microscope using a PLAPON 60 × 1.45 numerical aperture objective (Olympus, Japan). Fluorescence was excited by a 561 nm solid-state laser operated at a power of 25 mW. Images were recorded with an electron multiplying CCD (charge-coupled device) camera (CCD-C9100-13, Hamamatsu, Japan). In FRET experiments, color channels were separated by projecting donor and acceptor emission on different parts of the CCD chip using an image splitter (dual view micro imager DV2, Photometrics, USA), filter specifications HQ 605/40, HQ 680/30 (Chroma Technology). Movies were recorded at a rate of 30 frames per second. The experiments were carried out at 22 °C.

Data analysis

Fluorescence time courses for donor (Cy3) and acceptor (Cy5) were extracted using custom-made Matlab (MathWorks) software as described (5, 6). A semi-automated algorithm (Matlab) was used to select anti-correlated fluorescence traces (correlation coefficient <0.1) exhibiting characteristic single fluorophore intensities. The bleed-through of Cy3 signal into the Cy5 channel was corrected using an experimentally determined coefficient (~0.13 in our setup; (5)). Leakage of Cy5 fluorescence into the Cy3 channel was not detected. All trajectories were smoothed over three data points and truncated to remove photobleaching and photoblinking events. Traces with lifetimes of Cy3 or Cy5 less than 20 frames (0.66 s) or with multiple photobleaching steps were excluded from the analysis. The FRET efficiency was defined as the ratio of the measured emission intensities, Cy5/(Cy3+Cy5) (6). FRET time courses were fitted by Hidden Markov modeling using the vbFRET software package (http://vbfret.sourceforge.net/) (7). Models with different number of states were considered for each data set. FRET changes of <0.1 in idealized trajectories were not considered as transitions. Transitions lasting for only one frame were not included in the analysis as well. About 5% of all traces were poorly idealized by Hidden Markov modelling and eliminated from subsequent analysis. Two-dimensional contour plots were generated from time-resolved FRET trajectories. The set of all FRET traces for a given condition was compiled in a histogram, which was fitted to a sum of Gaussian functions using Matlab code (5). Mean FRET values (mean±sd) and population distribution (p=area under the curve±sd) were calculated from three independent datasets and are summarized in Table 1 and Table 3. Dwell times of different FRET states of fluctuating traces were extracted from idealized trajectories. The dwell time histogram for each transition was fitted to an exponential function, $y=y_0+Ae^{-t/\tau}$. Rates (k) were calculated by taking the inverse of dwell times (τ).

ATPase activity assay

ATP turnover by Prp43 was monitored using a coupled enzymatic assay following nicotinamide adenine dinucleotide (NADH) absorption at 340 nm over time in a VICTOR Nivo Multimode Microplate Reader (PerkinElmer) (8). Triplicate measurements were performed at room temperature in 20 mM Tris/HCl (pH 7.5), 150 mM KCl and 3 mM $MgCl_2$,250 nM NADH, 500 nM phosphoenolpyruvate, 6–8.3 U/ml pyruvate kinase and 9–14 U/ml lactic dehydrogenase. The ATP concentration ranged between 0 to 2 mM. To obtain suitable reaction velocities, Prp43 was used at a concentration of 2 µM (no stimulation), 0.5 µM (stimulation with Pfa1(gp)) or 0.2 µM (stimulation with Pfa1(gp) and RNA). For measurements in the presence of Pfa1(gp) a 5-fold molar excess over Prp43 was used.

Measurements in the presence of RNA were conducted with a 5-fold molar excess of an A_{20}-ssRNA (AXOlabs) over Prp43. The ATP consumption per minute (k_{obs}) was calculated using

$$k_{obs} = \frac{[\frac{\Delta A_{340}}{\Delta t}]}{\varepsilon_{340} \cdot d \cdot c}$$

where $\Delta A_{340}/\Delta t$ is the slope of the NADH decrease, ε_{340} is the extinction coefficient of NADH, *d* is the optical pathlength and *c* is the protein concentration. K_M and k_{cat} ($\frac{V_{max}}{[E]}$) were calculated by fitting the experimental data with the Michaelis-Menten equation

$$v = \frac{V_{max} \cdot [S]}{K_M + [S]}$$

where v is the reaction velocity, V_{max} is the maximal velocity of the system [S] is the substrate concentration and [E] is the total Prp43 concentration, using OriginPro 9.1.

Isothermal titration calorimetry (ITC)

The binding affinity of ctPfa1(gp) to ctPrp43 was measured by ITC with a MicroCal VP-ITC (Malvern Panalytical) using a concentration of 4 µM Prp43 in the cell and 53 µM Pfa1(gp) in the syringe. The reaction buffer contained 20 mM Tris/HCl (pH 7.5), 200 mM NaCl, 5% (v/v) glycerol and 2 mM $MgCl_2$. The initial injection of 5 µl was followed by 15 µl injections performed at a speed of 0.5 $µls^{-1}$ with intervals of 360 s between injections. The reference energy was set to 10 $µCals^{-1}$ and the binding was monitored at 25 °C. Stoichiometry of binding and dissociation constant were determined in three independent experiments, data analysis was carried out using MicroCal VP-ITC Analysis software (Malvern Panalytical).

RNA binding assay

The RNA binding of Prp43 was measured by fluorescence polarization spectroscopy using a VICTOR Nivo Multimode Microplate Reader (PerkinElmer). The binding of 6 nM 3′ 6-carboxyfluorescein-labeled A_{20}-RNA (Sigma Aldrich) to up to 100 µM scPrp43 was monitored as triplicates at room temperature in 20 mM Tris/HCl (pH 7.5), 200 mM NaCl, 5% glycerol and 3 mM $MgCl_2$. For measurements in presence of Pfa(gp), the complex was formed by adding a 5-fold molar excess over Prp43 and incubating for 10 min at room temperature. Experiments in presence of ADP or AMPPNP were performed at a constant concentration of 3.5 mM throughout all measurements. The excitation wavelength was 480 nm and the emission was detected at 530 nm for 500 ms. The data were normalized by setting the maximum of measured polarization to 100% and the polarization measured without the addition of protein to 0%. The data were fitted by nonlinear regression with a sigmoidal dose response equation

$$r = r_0 + \frac{\Delta r_{max}}{1 + (\frac{[E]_T}{K_D})^p}$$

where r is the measured polarization, r_0 the initial polarization, Δr_{max} the maximum amplitude of polarization, $[E]_T$ the total protein concentration and K_D the dissociation constant, using the analysis software OriginPro 9.1.

ADP binding assay

Binding of ADP to Prp43 was measured by fluorescence polarization spectroscopy using a Fluorescence spectrophotometer FluoroMax 3 (Horiba Scientific). The binding of 100 nM mant-labeled ADP (Jena Bioscience) to up to 20 µM ctPrp43 was monitored as triplicates at room temperature in 20 mM Tris/HCl (pH 7.5), 200 mM NaCl, 5% glycerol and 3 mM $MgCl_2$. For measurements in presence of Pfa(gp) and ssRNA the complex was formed by adding a 5-fold molar excess over Prp43 and incubating for 10 min at room temperature. The excitation wavelength was 360 nm and the emission was detected at 450 nm. The data were fitted by nonlinear regression as detailed above.

ADP release assay

ADP release from ctPrp43 was followed through FRET, with Prp43's tryptophan residues acting as the FRET donor, and the mant-label on the nucleotide as the FRET acceptor. For the determination of dissociation rates (k_{off}), 10 µM of labelled nucleotide was preincubated for 10min at room temperature with 1 µM ctPrp43 in 20 mM Tris/HCl (pH 7.5), 200 mM NaCl, 5% glycerol and 3 mM $MgCl_2$, in presence or absence of a 5-fold molar excess of ctPfa1(gp) or A_{20} RNA. The sample was rapidly mixed with 1 mM unlabeled "dark" ADP using a Stopped Flow Spectrometer SX20 (Applied Photophysics). The excitation wavelength was set to 280 nm and the emission of the mant-label was detected at 450 nm using a 395 nm filter to eliminate stray light. The reaction was followed for 60 to 1800 s and the time curves were fitted to a one phase exponential function using the analysis software OriginPro 9.1.

RNA unwinding assay

RNA duplex unwinding by Prp43 was monitored by fluorescence measurements. The substrates contained a 12-nt single-stranded 3′- overhang and duplexes ranging from 10-20 base pairs. The short strand was labeled with Atto 488 at the 3′-end, while an Eclipse Quencher was attached to the 5′-end of the long strand.

Q-20: 5′-(Eclipse)GCCUACG GUGGUG CGUAGGC AAAAAAAAAAAA-3′
D-20: 5′-GCCUACG CACCAC CGUAGGC-(Atto488)-3′
Q-18: 5′-(Eclipse)CCUACG GUGGUG CGUAGG AAAAAAAAAAAA-3′
D-18: 5′-CCUACG CACCAC CGUAGG-(Atto488)-3′
Q-16: 5′-(Eclipse)CCUACG UGGU CGUAGG AAAAAAAAAAAA-3′
D-16: 5′-CCUACG ACCA CGUAGG-(Atto488)-3′
Q-14: 5′-(Eclipse)CUACG UGGU CGUAG AAAAAAAAAAAA-3′
D-14: 5′-CUACG ACCA CGUAG-(Atto488)-3′
Q-10: 5′-(Eclipse)ACG UGGU CGU AAAAAAAAAAAA-3′
D-10: 5′-ACG ACCA CGU-(Atto488)-3′

For annealing, the strands were mixed at a ratio of 1:1.2 (dye:quencher), incubated at 95 °C for 5 min and subsequently cooled to room temperature.
To monitor unwinding, 1 nM of RNA substrate was preincubated for 10min at room temperature with 500 nM ctPrp43 in 20 mM Tris/HCl (pH 7.5), 150 mM KCl and 3 mM MgCl2, in presence or absence of a 5-fold molar excess of ctPfa1(gp). The sample was rapidly mixed with 4 mM ATP or 4 mM ATP supplemented 100 µg/ml Poly U RNA (trap RNA) using a Stopped Flow Spectrometer SX20 (Applied Photophysics). The fluorescence of the Atto 488 dye was exited at 470 nm and the emission was detected using a 500 nm cutoff filter (KV 500, Schott). The reaction was followed for 20-40s. Strand separation is reported by

dequenching of the ATTO488 fluorophore. The separated single strands form internal stem loops to prevent reannealing. The presence of trap RNA prevented repeated unwinding attempts by binding Prp43 after dissociation from the duplex substrate.
The maximum amplitudes of the fluorescence time traces in absence of trap RNA correspond to complete unwinding of the duplex constructs. The fluorescence time traces in presence of trap RNA were normalized by division by the respective maximum amplitude of the construct. Then the maximum amplitudes of the normalized fluorescence time traces were used to indicate the fraction of unwound RNA.
The length of the lag phase was defined as the time between the start of the experiment (t_0) and the minimum fluorescence value preceding the major fluorescence increase of the time course.

Supplementary Figures

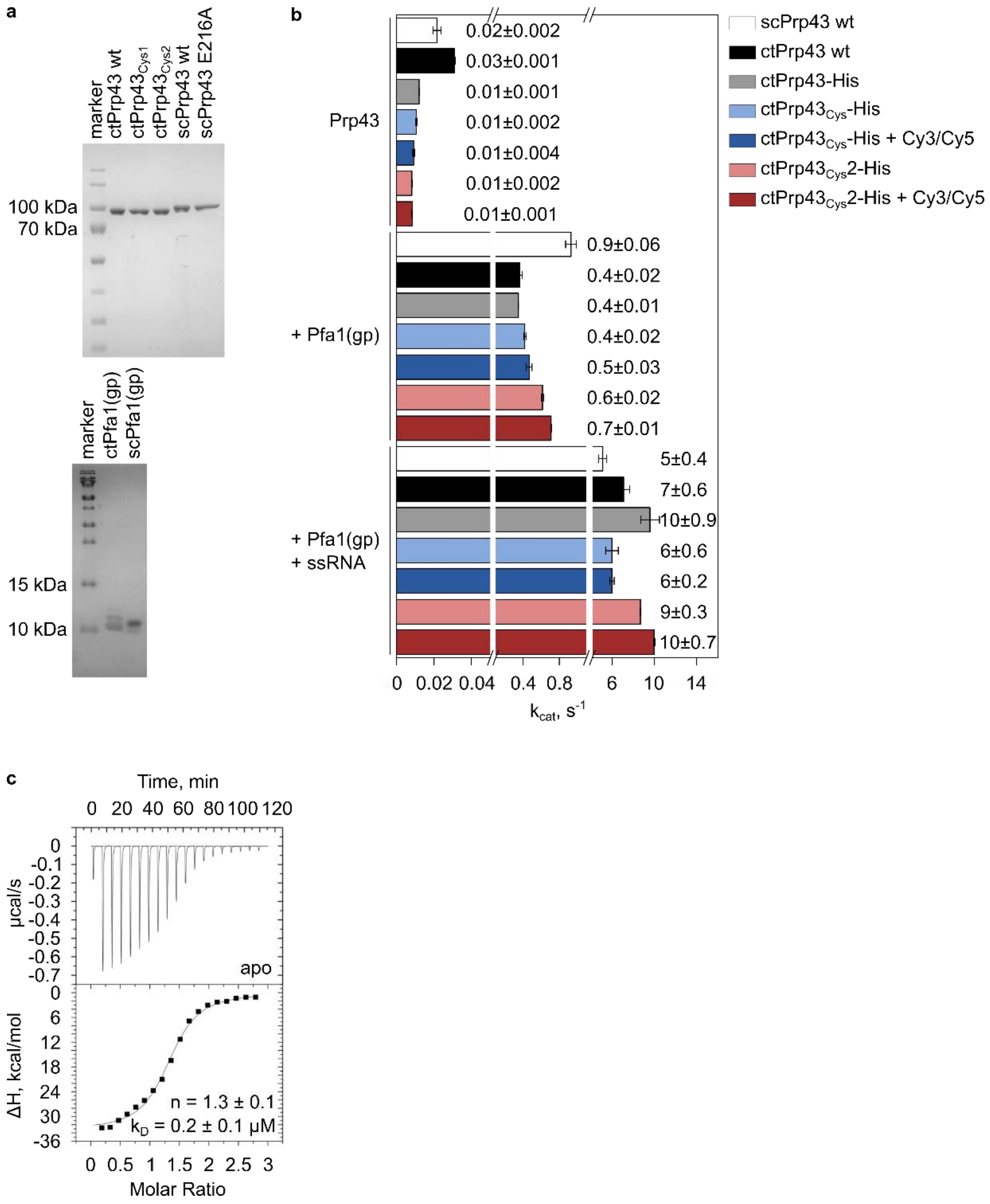

Supplementary Figure S1: ATPase activity and binding affinity of the Prp43-Pfa1(gp) complex.

a SDS-PAGE assessing the purity of ctPrp43, scPrp43 (wt and variants, 10 % acrylamide gel, above), ctPfa1(gp) and scPfa1(gp) (17.5 % acrylamide gel, below). A protein ladder ranging from 10-180 kDa (Thermo) is shown as reference.

b Steady-state ATP hydrolysis rates of scPrp43, ctPrp43 with and without His-tag, mutant ctPrp43 ($Prp43_{Cys}$ and $Prp43_{Cys}2$) with reactive cysteines in RecA1 and RecA2, and Cy3/Cy5 labeled $Prp43_{Cys}$ and $Prp43_{Cys}2$ at 2 mM ATP. The labeling efficiency of $Prp43_{Cys}$ was 77% and 66% for Cy3 and Cy5, respectively. $Prp43_{Cys}2$ was labeled by 80% with Cy3 and by 81% with Cy5. Pfa1(gp) and A_{20}-ssRNA were added at saturation. k_{cat}±sd were obtained in N=3 independent measurements.

c Representative binding titration of Prp43 with Pfa1(gp) monitored by isothermal titration calorimetry (ITC). Cell and syringe contained 4 µM Prp43 and 53 µM Pfa1(gp). Stoichiometry of binding (n=1.3±0.1) and dissociation constant (K_D=0.19±0.02 µM) were determined in N=3 independent experiments.

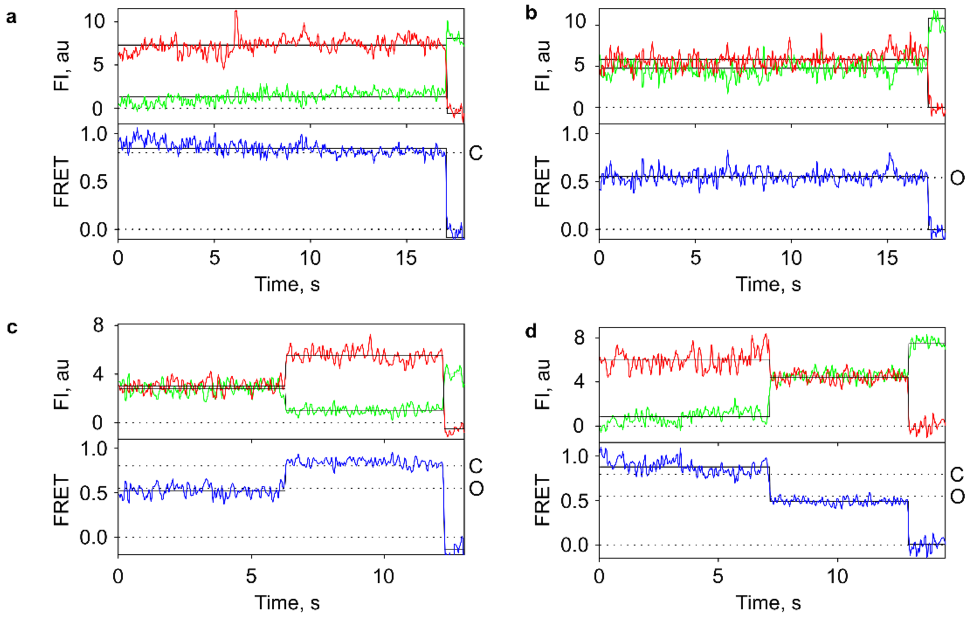

Supplementary Figure S2: smFRET signals of Cy3/Cy5 labeled Prp43$_{Cys}$.

a-d Representative time traces of Cy3- (green) and Cy5- (red) fluorescence intensity (FI) and FRET (blue, bottom plots) corresponding to (**a**) closed (C) RecA domains with E_{FRET}=0.8 or (**b**) open (O) RecA domains with E_{FRET}=0.55 and to transitions (**c**) from the O to C or (**d**) from the C to O state. Solid lines represent the Hidden-Markov fit of the traces.

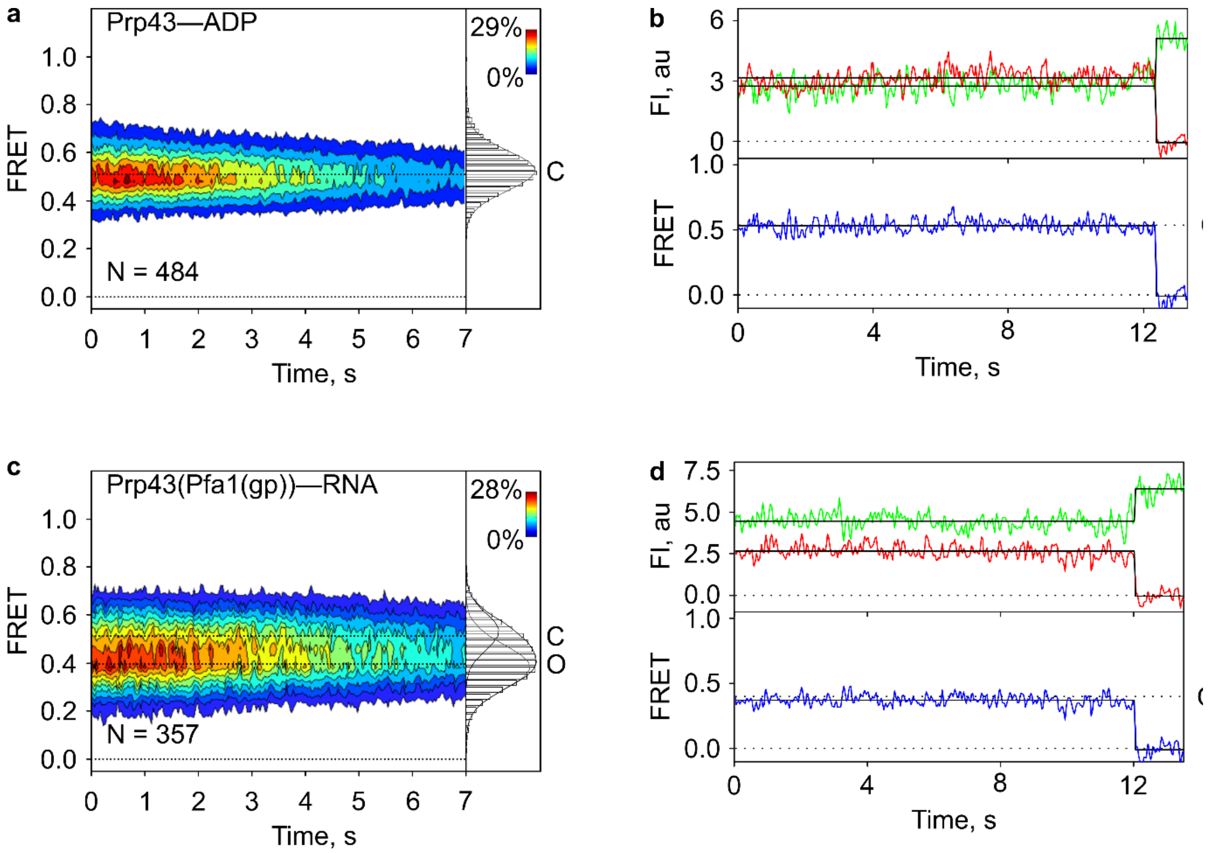

Supplementary Figure S3: smFRET signals of labeled Prp43$_{Cys}$2.

a and **c** Contour plots and 2D histograms showing the distribution of FRET values (mean±sd, derived from N=3 independent data sets) of (**a**) Prp43$_{Cys}$2—ADP (0.53±0.02) and (**c**) Prp43-Pfa1(gp)-RNA (0.56±0.04and 0.40±0.01).

b and **d** Representative time traces of Cy3- (green) and Cy5- (red) fluorescence intensity (FI) and FRET (blue, bottom plots) corresponding to (**b**) C and (**d**) O state of Prp43$_{Cys}$2. Solid lines represent the Hidden-Markov fit of the traces.

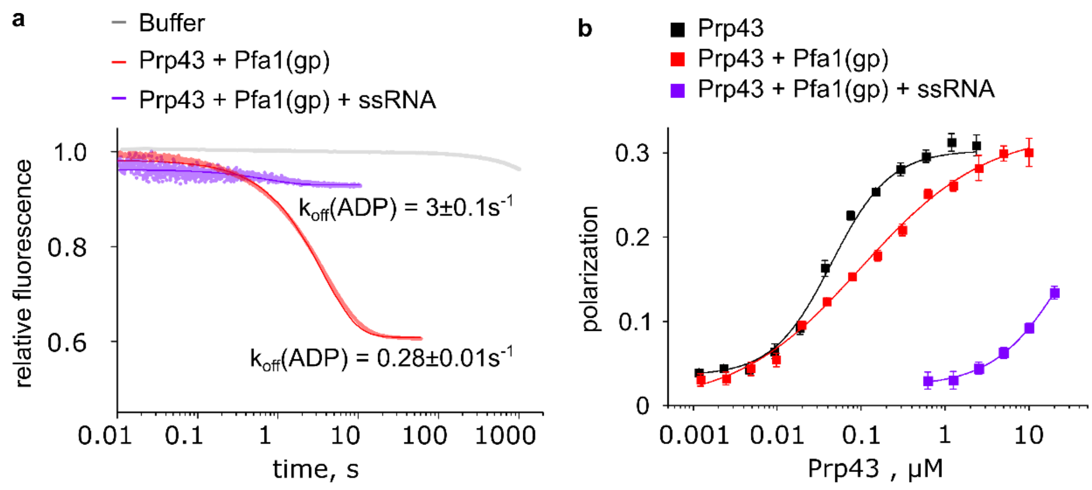

	**K_D(ADP), µM**
Prp43	0.04±0.003
Prp43 + Pfa1(gp)	0.1±0.01
Prp43 + Pfa1(gp) + ssRNA	> 20

Supplementary Figure S4: Binding and release of mant-ADP.

a Release of mant-ADP from Prp43 monitored by stopped-flow. Averages of fluorescence time traces, normalized by fluorescence intensity, are shown for Prp43-Pfa1(gp) and Prp43-Pfa1(gp)—ssRNA. Dissociation rates (k_{off}(ADP)±sd) are indicated in the plot. Due to the weak binding of mant-ADP to Prp43 in presence of Pfa1(gp) and ssRNA the amplitude of the corresponding time trace is very low.

b mant-ADP binding affinity to Prp43 determined by fluorescence polarization spectroscopy in the presence of Pfa1(gp) (red) or Pfa1(gp) and ssRNA (purple). Shown are mean values; error bars correspond to the sd derived from N=3 independent measurements. The table indicates affinity constants (K_D(mant-ADP)±sd). In presence of Pfa1(gp) and ssRNA less than 50% polarization was reached, the indicated K_D is an estimate of the lower limit.

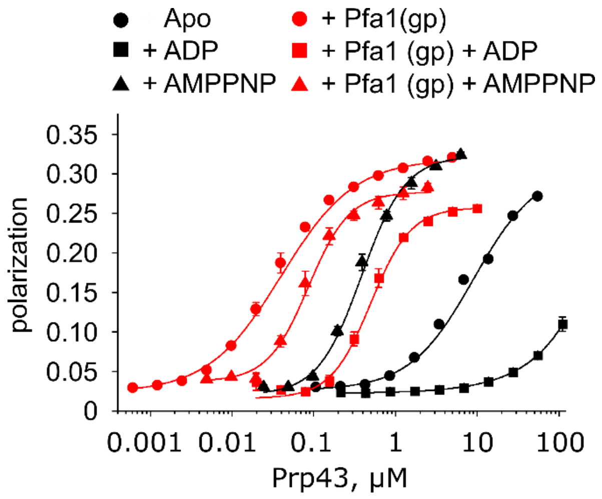

Supplementary Figure S5: Binding of scPrp43 to ssRNA (raw data).
scPrp43 binding affinity to ssRNA determined by fluorescence polarization spectroscopy in the absence (black) or presence (red) of Pfa1(gp) in the nt-free (circles), ADP- (squares) or AMPPNP- (triangles) state. Shown are mean values of raw data; error bars correspond to the sd derived from N=3 independent measurements.

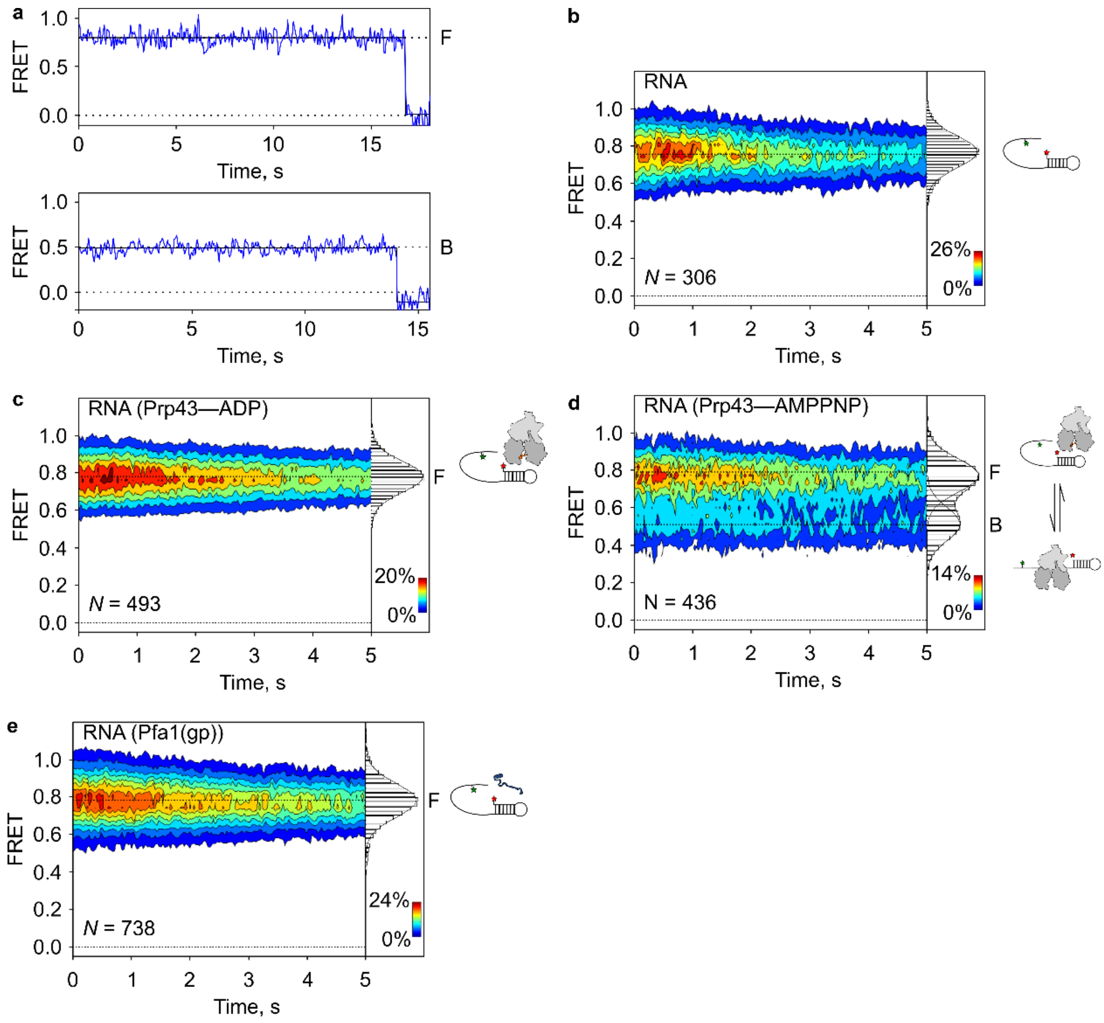

Supplementary Figure S6: Assignment of conformational states of the RNA probe.

a Representative smFRET time traces corresponding to the F (top plot) and B (bottom plot) RNA states. Solid lines represent the Hidden-Markov fit.

b-e Contour plots and 2D histograms of FRET values (mean±sd, derived from N=3 independent data sets) of the RNA probe (**b**) in isolation (0.79±0.01), (**c**) in the presence of 5 µM Prp43 and 2 mM ADP (0.78±0.02), (**d**) in the presence of 0.5 µM Prp43 and 2 mM AMPPNP (0.79±0.01, 0.52±0.02) and (**e**) in the presence of 2.5 µM Pfa1(gp) (0.80±0.01).

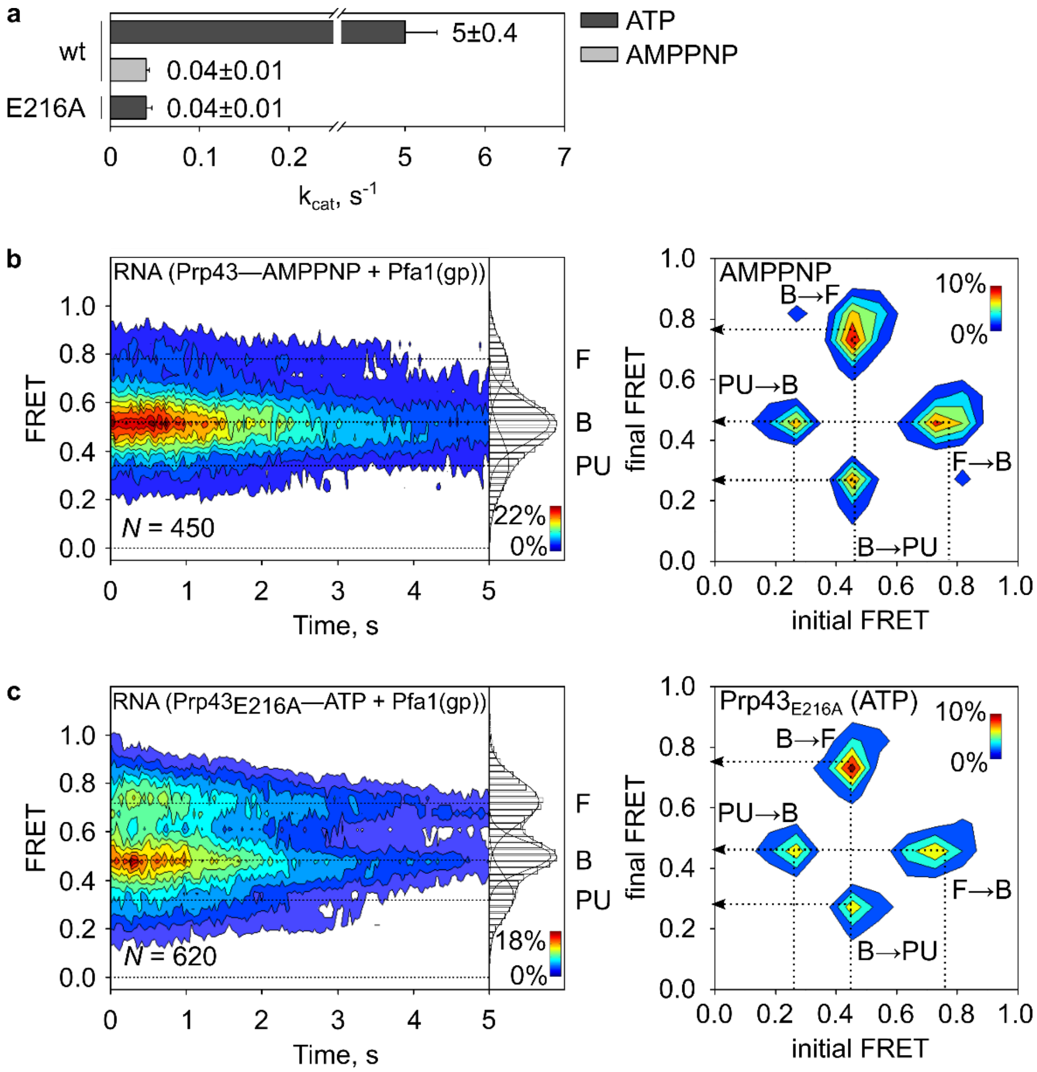

Supplementary Figure S7: Unwinding of dsRNA in the absence of continuous ATP hydrolysis.

a Steady-state ATP hydrolysis rates of $scPrp43_{wt}$ at 2mM ATP or 2 mM AMPPNP and $scPrp43_{E216A}$ at 2 mM ATP. Pfa1(gp) and A_{20}-ssRNA were added at saturation. k_{cat}±sd were obtained in N=3 independent measurements.

b Contour plot and 2D histogram showing the distribution of FRET values (mean±sd, derived from N=3 independent data sets) of the RNA probe in the presence of the Prp43-Pfa1(gp) complex at 2mM AMPPNP (0.77±0.02 (F), 0.51±0.02 (B), and 0.28±0.05 (PU)) and corresponding transition density plot visualizing the frequency of transitions between B and F or B and PU states.

c Contour plot and 2D histogram showing the distribution of FRET values (mean±sd, derived from N=3 independent data sets) of the RNA probe in the presence of the $Prp43_{E216A}$-Pfa1(gp) complex at 2mM ATP (0.74±0.02 (F), 0.50±0.01 (B) and 0.34±0.04 (PU)) and corresponding transition density plot visualizing the frequency of transitions between B and F or B and PU states.

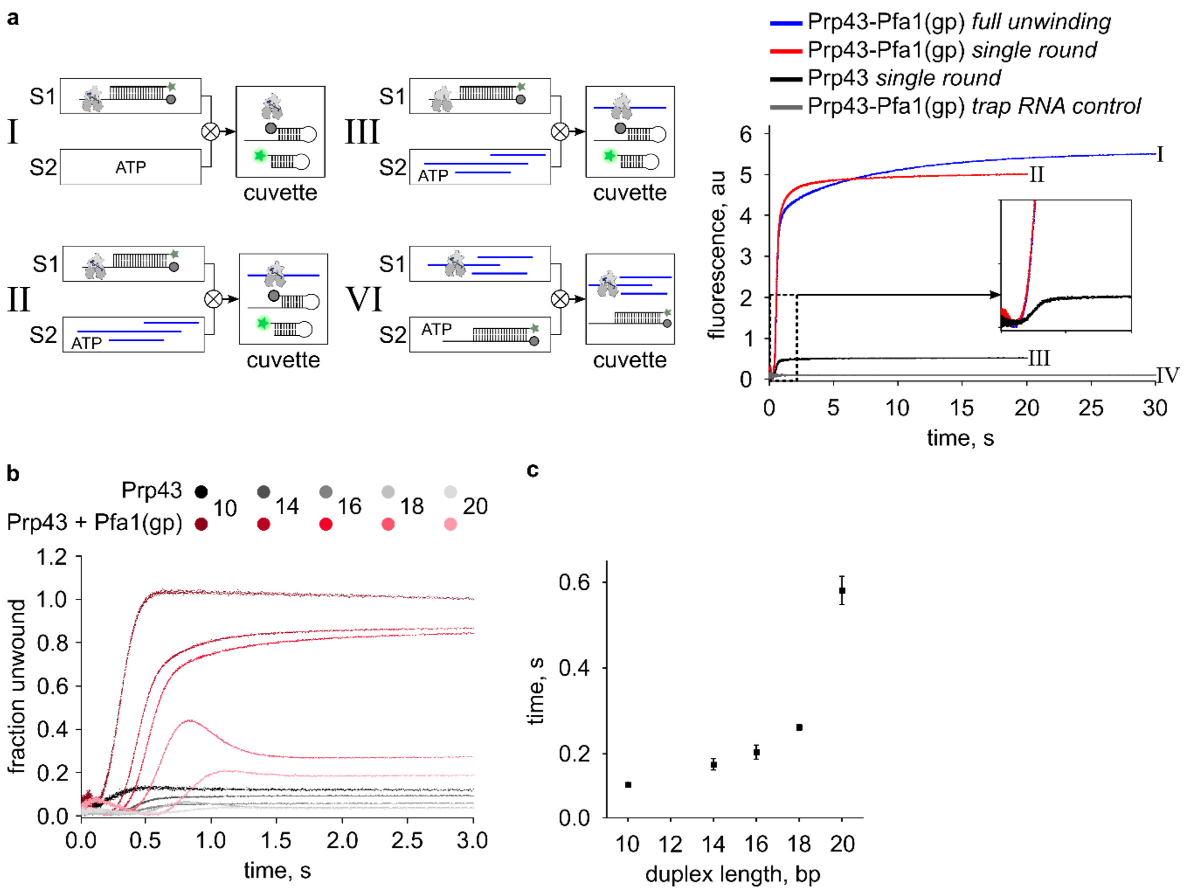

Supplementary Figure S8: Unwinding of duplex RNA monitored by stopped-flow.

a Stopped-flow experiment scheme and time course of fluorescence changes during unwinding of the 16 bp duplex by Prp43 or the Prp43-Pfa1(gp) complex. Inset shows zoom-in into the first 2 seconds of the time course. I, blue trace: Unwinding by Prp43-Pfa1(gp) complex at multiple round conditions. Syringe 1 (S1) contains the duplex RNA construct labeled by ATTO488 (green star) and Eclipse quencher (grey circle) preincubated with Prp43-Pfa1(gp) complex. Syringe 2 (S2) contains 4 mM ATP. Upon rapid mixing the separation of duplex strands becomes apparent by dequenching of the ATTO488 fluorophore (bright green star). Single strands fold into stem-loops to prevent reannealing. II, red trace: Unwinding by Prp43-Pfa1(gp) complex at single round conditions. S1 contains the labeled duplex RNA preincubated with Prp43-Pfa1(gp) complex, while S2 contains PolyU RNA (100 µg/ml) and ATP. III, black trace: Unwinding by Prp43 at single round conditions. S1 contains the labeled duplex RNA preincubated with Prp43, S2 contains PolyU RNA and ATP. IV, grey trace: S1 contains Poly U RNA preincubated with Prp43-Pfa1(gp) complex, S2 contains the labeled duplex RNA and ATP. The excess of trap RNA does not allow binding and unwinding of the duplex. This indicates that it binds efficiently to Prp43 which is prerequisite for single round RNA unwinding.

b Time course of RNA duplex unwinding by Prp43 (grey scale time traces) and by Prp43-Pfa1 complex (red time traces) at single round conditions. Data were normalized by division by the maximum amplitude of the fluorescence time course corresponding to the complete unwinding of RNA duplexes obtained by repeating the experiments at multiple round conditions (see (a)). Shown are averaged time traces from at least three technical replicates.

c Length of the lag phase corresponding to the time between t_0 and the minimum fluorescence value preceding the major fluorescence increase of the time course depending on duplex length. Shown are mean values and error bars corresponding to the sd derived from N=3 independent measurements.

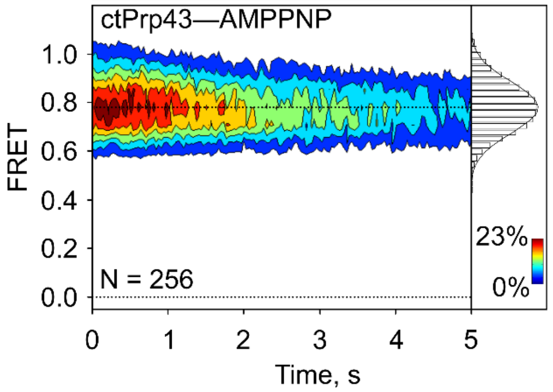

Supplementary Figure S9: RecA domain conformation in presence of AMPPNP.
Contour plot and 2D histogram showing the distribution of FRET values (mean±sd, derived from N=3 independent data sets) of Cy3-/Cy5-labeld Prp43—AMPPNP (0.79±0.01).

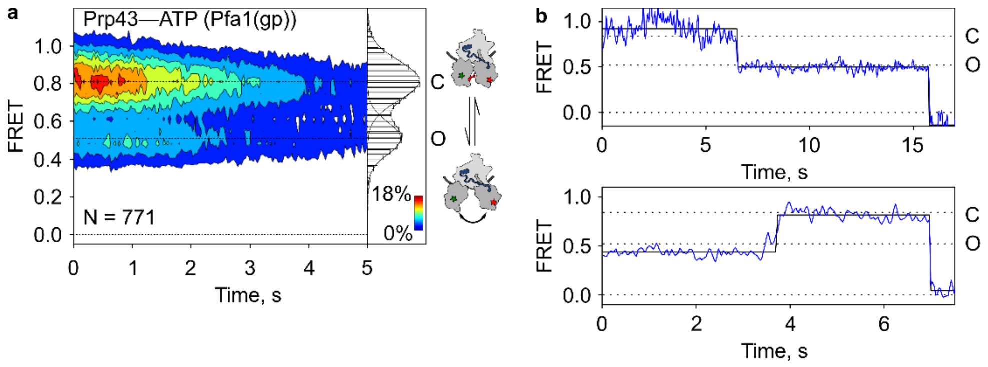

Supplementary Figure S10: Acceleration of conformational cycling by Pfa1(gp).

a Contour plot and 2D histogram showing the distribution of FRET values (mean±sd, derived from N=3 independent data sets) of Cy3-/Cy5-labeld Prp43—ATP in complex with Pfa1(gp) (0.83±0.02 and 0.51±0.01).

b Representative FRET traces of Cy3-/Cy5-labeld Prp43—ATP in complex with Pfa1(gp) showing transitions from C to O (above) and from O to C (below) states.

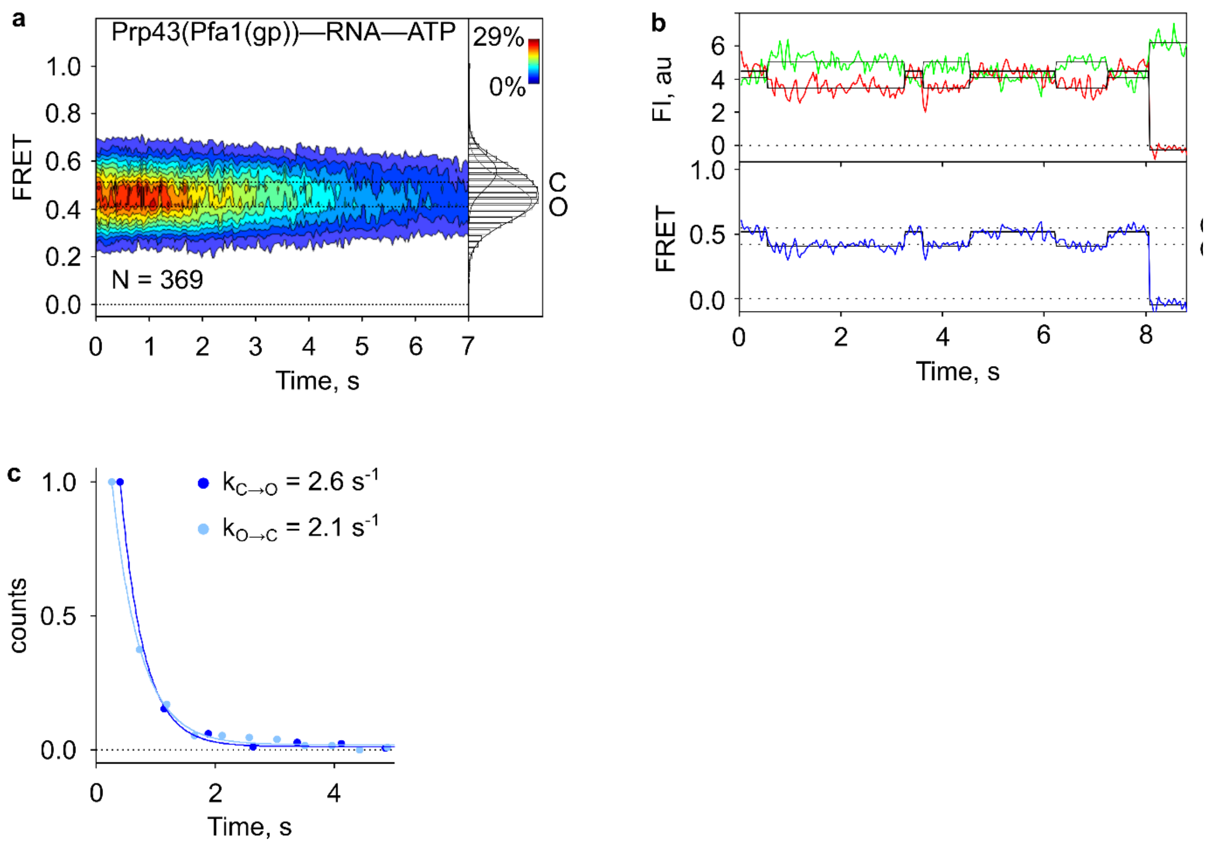

Supplementary Figure S11: Conformational cycling of Prp43$_{Cys}$2 during ATP hydrolysis.

a Contour plot and 2D histogram showing the distribution of FRET values (mean±sd, derived from N=3 independent data sets) of Cy3-/Cy5-labeld Prp43$_{Cys}$2-Pfa1(gp)-RNA—ATP (0.55±0.02 and 0.42±0.01).

b Representative time trace of Cy3- (green) and Cy5- (red) fluorescence intensity (FI) and FRET (blue, bottom plot) of Cy3-/Cy5-labeld Prp43$_{Cys}$2-Pfa1(gp)-RNA—ATP, showing multiple transitions between C and O state.

c The distributions of dwell times of the C (dark blue) and O (light blue) states, normalized by the number of FRET counts, were fitted by an exponential function to determine the transition rates between the states. n=240 and n=235 transitions for C→O and O→C respectively were included in the analysis.

a

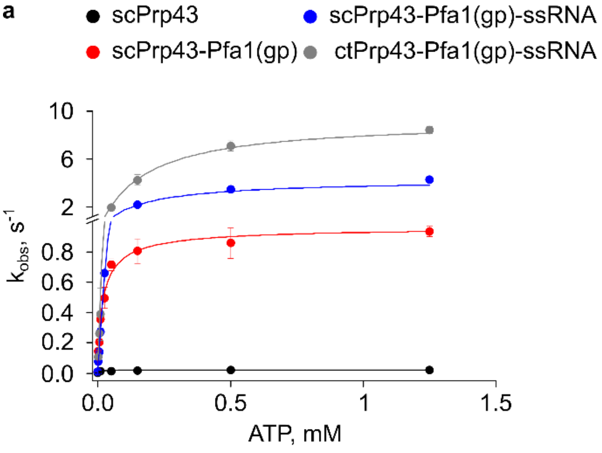

	k_{cat}, s^{-1}	K_M, µM
scPrp43	0.02±0.002	11.5±6
ctPrp43	0.012±0.001	29.5±6
scPrp43 + scPfa(gp)	0.9±0.06	18.0±1
ctPrp43 + ctPfa(gp)	0.35±0.02	4.5±0.3
scPrp43 + scPfa(gp)/ssRNA	5.1±0.4	210±42
ctPrp43 + ctPfa(gp)/ssRNA	9.6±0.9	191±60

Supplementary Figure S12: Comparison of ATPase activity between scPrp43 and ctPrp43. Michealis-Menten titration and table with ATP turnover numbers (k_{cat}) and the Michaelis-Menten constants (K_M) showing the stimulation of scPrp43 ATPase activity by scPfa1(gp) and ssRNA compared to ctPrp43 stimulated by ctPfa1(gp) and ssRNA.

Supplementary Table 1: Analysis of RecA domain conformation.

	N	$FRET_C$	$FRET_O$	P_C/P_O	Dyn
**Steady-state**					
Prp43	292	0.77±0.02			
Prp43 + Pfa1(gp)	521	0.75±0.05	0.56±0.03	0.66±0.08/0.34±0.08	7%
Prp43—ADP	694	0.81±0.02			
$Prp43_{Cys}2$—ADP	484	0.53±0.02	-	-	-
Prp43—ADP + Pfa1(gp)	395	0.82±0.03	0.54±0.03	0.79±0.04/0.21±0.04	
Prp43—RNA	326	0.79±0.02	0.53±0.04	0.83±0.02/0.17±0.02	
Prp43—RNA + Pfa1(gp)	574	0.77±0.02	0.54±0.01	0.35±0.06/0.65±0.06	6%
$Prp43_{Cys}2$—RNA + Pfa1(gp)	357	0.56±0.04	0.40±0.01	0.26±0.07/0.74±0.07	5%
**ATP hydrolysis**					
Prp43—ATP	743	0.80±0.02	0.53±0.02	0.87±0.02/0.13±0.02	4%
Prp43—AMPPNP	256	0.79±0.01	-	-	-
Prp43—ATP + Pfa1(gp)	689	0.83±0.02	0.51±0.01	0.65±0.05/0.35±0.05	27%
Prp43—RNA—ATP + Pfa1(gp)	541	0.80±0.01	0.52±0.01	0.70±0.02/0.30±0.02	37%
$Prp43_{Cys}2$—RNA—ATP + Pfa1(gp)	369	0.55±0.02	0.42±0.01	0.31±0.10/0.69±0.10	42%

N – number of traces obtained in three independent measurements

FRET – mean of FRET distribution ± sd

P_C – fraction of population in the C state ± sd

P_O – fraction of population in the O state ± sd

Dyn – percentage of traces with C→O and O→C transitions

Supplementary Table 2: RecA Inter domain distances of Prp43 in C and O conformations.

	R_{D-A} (Förster Equation)	$R_{C\alpha}$ (Structural data)
**Closed RecA domains**		(Prp43-ADP, PDB: 5D0U)
$Prp43_{Cys}$	4.3 nm	4.2 nm
$Prp43_{Cys}2$	5.3 nm	5.2 nm
**Open RecA domains**		
$Prp43_{Cys}$	5.2 nm	5.0 nm (Prp22-RNA, PDB: 6I3P)
		5.1 nm (Prp43, PDB: 5y88)
$Prp43_{Cys}2$	5.8	5.9 nm (Prp22-RNA, PDB: 6I3P)
		6.0 nm (Prp43, PDB: 5y88)
**Difference**		
$Prp43_{Cys}$	0.9 nm	0.8-0.9 nm
$Prp43_{Cys}2$	0.5 nm	0.7-0.8

R_{D-A} – distance between Cy3 and Cy5 calculated using an inverse 6th-power law ($E(FRET)=1/(1+(r/R_0)^6)$) and a Förster Radius of $R_0 = 5.4$ nm (9, 10)

$R_{C\alpha}$ – distance between the C_α atoms of K170C and C303 in $ctPrp43_{Cys}$ and S187C and C303 in $ctPrp43_{Cys}2$ or their equivalents in scPrp43 (K167 or N184 and T300) and ctPrp22 (Q629 or P646 and T762)

Supplementary Table 3: FRET state analysis of the Cy3- and Cy5-labeled RNA substrate.

Protein factors added	N	$FRET_F$	$FRET_B$	$FRET_{PU}$	$P_F/P_B/P_{PU}$	Dyn
No protein	306	0.79±0.01				
Pfa1(gp)	406	0.80±0.01				
Prp43	180	0.77±0.04	0.49±0.02		0.79±0.05/ 0.21±0.05	4%
Prp43 + Pfa1(gp)	922	0.78±0.03	0.48 ±0.02		0.12±0.01/ 0.88±0.01	5%
Prp43—ADP	493	0.78±0.02				
Prp43—ADP + Pfa1(gp)	481	0.80±0.02	0.51±0.02		0.48±0.09/ 0.52±0.09	16%
Prp43—AMPPNP	436	0.79±0.01	0.52±0.02		0.64±0.04/ 0.36±0.04	11%
Prp43—ATP + Pfa1(gp)	576	0.76±0.03	0.51±0.01	0.35±0.08	0.24±0.12/ 0.55±0.07/ 0.2±0.08	20%
Prp43—AMPPNP + Pfa1(gp)	450	0.77±0.02	0.51±0.02	0.28±0.05	0.21±0.02/ 0.68±0.08/ 0.11±0.09	30%
$Prp43_{E216A}$—ATP + Pfa1(gp)	620	0.74±0.02	0.50±0.01	0.34±0.04	0.44±0.03/ 0.34±0.04/ 0.22±0.04	34%

N – number of traces obtained in three independent measurements

FRET – mean of FRET distribution ± sd

P_F – fraction of population in the free (F) state ± sd

P_B – fraction of population in the bound (B) state ± sd

P_{PU} – fraction of population in the partially unwound (PU) state ± sd

Dyn – percentage of traces with transitions between F, B and PU states

Supplementary Table 4: Transition frequency analysis.

**RNA binding**	**$k_{B \rightarrow F}$ (n), s^{-1}**	
Prp43—ADP + Pfa1(gp)	0.45 (76)	
**RecA domain conformation**	**$k_{O \rightarrow C}$ (n), s^{-1}**	**$K_{C \rightarrow O}$ (n), s^{-1}**
Prp43—ATP + Pfa1(gp)	0.47 (56)	0.90 (104)
Prp43—RNA—ATP + Pfa1(gp)	1.92 (223)	1.44 (228)
$Prp43_{Cys}2$—RNA—ATP + Pfa1(gp)	2.1 (235)	2.6 (240)

n – number of transitions

$k_{B \rightarrow F}$ – transition rate from the bound (B) to the free (F) state

$k_{C \rightarrow O}$/ $k_{O \rightarrow C}$ – transition rate from the closed (C) to the open (O) state and *vice versa*

SI References

1. M. J. Tauchert, J. B. Fourmann, H. Christian, R. Lührmann, R. Ficner, Structural and functional analysis of the RNA helicase Prp43 from the thermophilic eukaryote Chaetomium thermophilum. *Acta Crystallogr. Sect. Struct. Biol. Commun.* **72**, 112–120 (2016).
2. J.-B. B. Fourmann, M. J. Tauchert, R. Ficner, P. Fabrizio, R. Lührmann, Regulation of Prp43-mediated disassembly of spliceosomes by its cofactors Ntr1 and Ntr2. *Nucleic Acids Res.* **45**, 4068–4080 (2017).
3. F. W. Studier, Protein production by auto-induction in high density shaking cultures. *Protein Expr. Purif.* **41**, 207–234 (2005).
4. E. Gasteiger, *et al.*, "Protein Identification and Analysis Tools on the ExPASy Server" in *The Proteomics Protocols Handbook*, (2005), pp. 571–608.
5. S. Adio, *et al.*, Fluctuations between multiple EF-G-induced chimeric tRNA states during translocation on the ribosome. *Nat. Commun.* **6**, 1–11 (2015).
6. R. Roy, S. Hohng, T. Ha, A Practical Guide to Single Molecule FRET. *Nat. Methods* **5**, 507–516 (2008).
7. J. E. Bronson, J. Fei, J. M. Hofman, R. L. Gonzalez, C. H. Wiggins, Learning rates and states from biophysical time series: A Bayesian approach to model selection and single-molecule FRET data. *Biophys. J.* **97**, 3196–3205 (2009).
8. K. C. Agarwal, R. P. Miech, R. E. Parks, Guanylate Kinases from Human Erythrocytes, Hog Brain, and Rat Liver. *Methods Enzymol.* **51**, 483–490 (1978).
9. H. Son, W. Mo, J. Park, J. W. Lee, S. Lee, Single-Molecule FRET Detection of Sub-Nanometer Distance Changes in the Range below a 3-Nanometer Scale. *Biosensors* **10**, bios10110168 (2020).
10. T. Förster, Energiewanderung und Fluoreszenz. *Naturwissenschaften* **33**, 166–175 (1946).
